# Fast and selective reversed-phase high performance liquid chromatographic separation of UO_2_^2+^ and Th^4+^ ions using surface modified C_18_ silica monolithic supports with target specific ionophoric ligands†

**DOI:** 10.1039/d2ra07495h

**Published:** 2023-01-23

**Authors:** Aswanidevi Kongasseri, Thirumalai Madhesan, Suchashrita Mitra, C. V. S. Brahmananda Rao, Sivaraman Nagarajan, Pitchaiah Kancharlapalli Chinaraga, Prabhakaran Deivasigamani, Akhila Maheswari Mohan

**Affiliations:** a Department of Chemistry, School of Advanced Sciences, Vellore Institute of Technology (VIT) Vellore Tamil Nadu 632014 India akhila.maheswari@vit.ac.in prabhakaran.d@vit.ac.in +91-9600061952; b Fuel Chemistry Division, Materials Chemistry and Metal Fuel Cycle Group, Indira Gandhi Centre for Atomic Research (IGCAR) Kalpakkam Tamil Nadu 603102 India

## Abstract

Reprocessing nuclear-spent fuels is highly demanded for enhanced resource efficacy and removal of the associated radiotoxicity. The present work elucidates the rapid separation of UO_2_^2+^ and Th^4+^ ions using a reversed-phase high-performance liquid chromatographic (RP-HPLC) technique by dynamically modifying the surface of a C_18_ silica monolith column with target-specific ionophoric ligands. For the dynamic modification, four analogous aromatic amide ligands, *N*^1^,*N*^1^,*N*^3^,*N*^3^,*N*^5^,*N*^5^-hexa(alkyl)benzene-1,3,5-tricarboxamide (alkyl = butyl, hexyl, octyl, and decyl) as column modifiers were synthesized. The complexation properties and retention profiles of the amide-based column modifiers for the selective and sequential separation of UO_2_^2+^ and Th^4+^ ions were investigated. In addition, the selective separation of UO_2_^2+^ and Th^4+^ ions among the competitive ions of similar chemical properties were also studied. The ionophore immobilized C_18_ silica monolith columns demonstrated a varying degree of retention behavior for UO_2_^2+^ and Th^4+^ ions (UO_2_^2+^ is retained longer than Th^4+^ under all analytical conditions), eventually leading to rapid separations within a period of ≤5 min. A 0.1 M solution of 2-hydroxyisobutyric acid (HIBA, 1 mL min^−1^) served as the mobile phase, and the qualitative and quantitative assessment of the sequentially separated 5f metal ions was achieved through post-column derivatization reaction, using arsenazo(iii) as a post-column reagent (PCR; 1.5 mL min^−1^) prior to analysis using a UV-vis detector, at 665 nm (*λ*_max_). The developed technique was further evaluated by standardizing various analytical parameters, including modifier concentration, mobile phase pH, mobile phase flow rate, *etc.*, to yield the best chromatographic separation. Also, the conceptual role of alkyl chain length (in the modifier) on the retention behavior of the studied metal ions was evaluated for cutting-edge future applications.

## Abstract 2

Reprocessing nuclear-spent fuels is highly demanded for enhanced resource efficacy and removal of the associated radiotoxicity.

## Graphical abstract

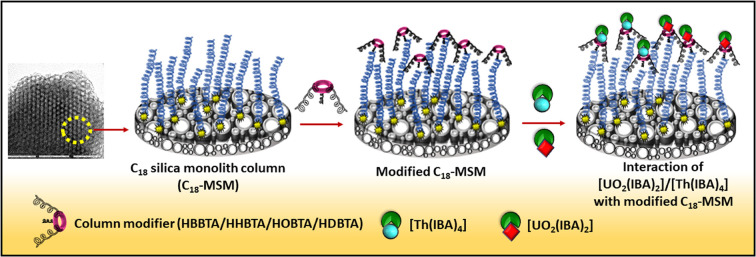

## 1. Introduction

With the manifold increase in the depletion rate of non-renewable energy resources, nuclear energy has evolved as one of the replacements in this domain of energy, and this coerces the opening up of the nuclear fuel cycle option, where a large amount of energy refining through nuclear reactors can be attained with critical and careful nuclear reprocessing of spent fuels.^1,2^ The utility of uranium and thorium as high-density penetrators, gyroscopic compasses, photographic chemicals, lamp filaments, contrast agents in medical radiography, ceramics, welding rods, camera and telescope lenses, fire brick, heat-resistant paint, *etc.*, promotes its urgent necessity of individual isolation from the crude sample sources.^3^ However, tracing and recovering these ions from environmental samples or other matrices is demanding due to the absence of accessible and specific methods.^4,5^ In this aspect, HPLC has gained much attention as a separation platform in the academic and industrial (preparative and process scale) sectors due to its rapid, robust, and reliable performance.^6^ In this line, LC-based monolith columns are emerging and find numerous applications where the quantity of analyte and the analysis times can be minimized tremendously for the best separation processes. Rapid and inexpensive analytical techniques for the detection and segregation of UO_2_^2+^ and Th^4+^ with great precision and high accuracy have already been reported in the literature. Several reported prevailing methods, such as solid-phase extraction (SPE), solvent extraction, ion exchange, precipitation, *etc.*, have been developed to separate UO_2_^2+^ and Th^4+^ from various matrices.^7–9^ Inductively coupled plasma–atomic emission spectrophotometry (ICP-AES) and ion-chromatography coupled to inductively coupled plasma mass spectrometry (IC-ICP-MS) are some of the sophisticated predominant methods in the sphere of analytical science for these targeted analytes.^10,11^ Although these techniques give appreciable detection limits, the shortcomings of these methods are their high-end instrumentation and operating costs with the requirement of skilled technicians. In this line, Dupuis *et al.* used capillary electrophoresis hyphenated with multi-collector inductively coupled plasma mass spectrometric technique for the isotope ratio measurements for actinides and lanthanides fission products from spent fuels.^12^ The technique can successfully lower the sample quantity and waste volume production. However, the cost factor associated with the instrumentation is far higher, which makes it less accessible for developing countries. The actinide separation process has also evolved using extraction chromatographic techniques coupled with ICP-MS, as described by Hang *et al.* Still, the method also suffers from high-end instrumental assay.^13^ Studies on the hydrometallurgical separation of trivalent actinides and lanthanides using CyMe_4_BTBP and CyMe_4_BTPhen extractants were also some recent alternatives for separating the concerned analytes.^14^ Wang *et al.* used simple extraction techniques to selectively separate actinides from lanthanides using a group of dithiophosphinic acids. The work mainly discusses the role of substituents in the dithiophosphinic acid on the extractive property and efficiency of the studied analytes, especially of Am^3+^ and Eu^3+^.^15^ There are also works reported using the ionic liquid hexyltributylphosphonium bis(trifluoromethylsulfonyl)imides,^16^ bis-1,2,3-triazolebipyridine ligands^17^ for the separation/extraction of lanthanides and actinides. The methods related to nanotechnology for the separation of actinides from radioactive waste are also developed. In line with this, Kaur *et al.* used magnetic nanoparticles (MNPs) conjugated with actinide-specific chelators to separate Np, Am, and *Cm* from spent nuclear fuels. The method is cost-effective, but the issues associated with the created secondary nanotoxicity need to be addressed.^18^ Also, extractions of minor actinides and lanthanides were studied using ligand immobilized on silica gel and using this method, near-quantitative removal of Am(iii) in the presence of Eu(iii) has been achieved by Afsar *et al.*^19^ Most of the method prevails till now either comes under sophisticated instrumental procedures which demand high costs or lags in specific removal of UO_2_^2+^/Th^4+^ amidst larger concentrations of lanthanide ions.

In addition, several radioanalytical techniques, such as isotope dilution analysis, radiochromatography, neutron activation analysis *etc.*, were reported for the isotopic detection of various radionuclides.^20,21^ The detection of these radionuclides also includes various environmental and biological samples. In this line, Roane *et al.* used a radiochromatographic technique to analyze actinides and strontium from soil samples.^22^ Quite recently, Jia *et al.* used radioanalytical techniques to separate and measure some radionuclides in the area of the Gela Phosphate Industry in Italy. The radionuclides ^238^U, ^234^U, ^235^U, ^226^Ra, ^210^Po, and ^216^Po were counted using α-spectrometry, and the techniques used were found to be sensitive, selective and accurate.^23^ The α-particle spectrometric technique was recently used for determining uranium and thorium by Lindahl *et al.*, which illustrates an alkaline fusion method for a low-cost total dissolution of soil and sediment samples containing U and Th isotopes.^24^ Similarly, Harrison *et al.* studied the separation of thorium, plutonium, americium, uranium and strontium in environmental matrices using radioanalytical techniques where the process involves sample dissolution, concentration *via* calcium phosphate co-precipitation, rapid column extraction using TEVA™, TRU™ and Sr-Spec™ resin cartridges and alpha spectrometry for Th, Pu, U and Am and Cerenkov counting for Sr.^25^ Besides, radioanalytical determination of uranium has been done using reactive polymer thin films. α-Spectroscopy pulse height spectra were analyzed and utilized in the work to study the role of selective layer film thickness on peak energy resolution.^26^ Besides, Fullmer *et al.* utilized a hybrid extractive scintillator resin for the detection of plutonium where the extractive scintillating resin was comprised of a silica base, functionalized for plutonium extraction, grafted with a plastic scintillator of polyvinyl toluene (PVT) and 2-(1-naphthyl)-4-vinyl-5-phenyloxazole (vNPO) fluor.^27^ Benedik *et al.* utilized neutron activation analysis for the ultra-trace determination of U and Th in electrolytic copper *via* their induced nuclides ^239^U/^239^Np and ^233^Pa, respectively.^28^

Monolithic silica columns possess high surface area and uniform mesoporous structures with orderly arranged inter-connected through-pores, making them the ideal candidates for chromatographic applications.^29^ The unique characteristics of these columns render high performance in separation speed, low column back pressure, and enhancement in mass transfer kinetics.^30,31^ Thus, HPLC coupled with a UV-vis detection system is utilized to separate UO_2_^2+^ and Th^4+^ ions amidst a mixture of lanthanides (Lns) to reduce the cost factor significantly. As per the reports on actinides' separation and extraction chemistry, amide-based aliphatic extractants have been used to recover actinides.^32,33^ The classic examples include long-chain amides of *n*-octyl(phenyl)(*N*,*N*-diisobutylcarbamoyl)methyl phosphine oxide (CMPO), tri-butyl phosphate (TBP) and *N*,*N*,*N*,*N*-tetraoctyl diglycolamide (TODGA) as extractants for the selective recovery of actinide ions from matrix constituents.^34–38^ The derivative of TODGA has been extensively used for analyzing the extraction behavior of various f-block elements, as these aliphatic amides were found to bind the targets strongly with reasonable stability. An ion-pair HPLC technique has been reported by Sivaraman *et al.* for the individual isolation of Lns from uranium, plutonium, and other fission products where the pre-separation of Ln-fission products has been achieved using di-(2-ethylhexyl) phosphoric acid coated Amberlite XAD-7 packed bead glass column, using camphor-10-sulfonic acid and α-hydroxy isobutyric acid as the mobile phases.^39^ The same research group used the camphor-10-sulfonic acid modified column to separate Lns using HIBA as the mobile phase through ion interaction chromatography.^40^ Even though these techniques qualify in the isolation process, the inhomogeneity in the packed bead columns and the resulting high back pressure significantly increases the plate height, ultimately leading to longer analysis times for industrial scale-up applications. These ion-pair chromatographic techniques follow a separation protocol that involves the coulombic interaction between the target ion complex and the ionic/charged support surface. However, their limitations stand by the fact that the surfactant adsorption process is significantly slow, which initiates the use of nearly 20 column volumes to obtain the required mobile phase/eluent composition. There were some studies devoted to the use of aliphatic amides to separate some or the whole of the f-block metal ions. The estimation of UO_2_^2+^ amidst a multi-fold concentration of Th^4+^ was reported by Vidyalakshmi *et al.* using dialkyl amide-modified C_18_ silica supports is one among them.^41^ Likewise, Raju *et al.* studied the retention behavior of uranium and thorium in the presence of Lns on aliphatic amide(s) modified reverse phase column for the separation of Th^4+^ and UO_2_^2+^ even under high Ln concentrations.^42^ Similar studies were also conducted by Raut *et al.*, which were based on both ion exchange and reverse phase behavior of 2,6-pyridine dicarboxylic acid for the complexation of UO_2_^2+^ and Th^4+^.^43^ In addition, Datta *et al.* have conducted extensive studies on the chromatographic separation of UO_2_^2+^ and Th^4+^ using short columns with small particle sizes to separate UO_2_^2+^ from Pu^3+^ and Pu^4+^.^44^ Inspired by the results obtained, they have used monolithic supports modified with bis-2-ethylhexyl succinamic acid as a stationary phase for burn-up measurements of fast reactor fuels.^45^ Quite differently, the bistriazinyl pyridine-coated reverse phase column has been used as a stationary phase by Deepika *et al.* to separate uranium, thorium, americium and Lns where nitric acid and α-hydroxy isobutyric acid (α-HIBA) was used as the mobile phases.^46^ Furthermore, Raut *et al.* described the RP-HPLC separation of Lns, Th^4+^ and UO_2_^2+^ using *n*-octane sulfonate as a column modifier that exhibited partial cation exchanger property and hydrophobicity.^47^ These methods and processes gave insights into the beautiful separation chemistry of chemically similar f-block elements. However, they lag due to the technical difficulties in reproducibility, the requirements of larger concentrations of the modifiers to yield the separations and high column back pressure due to the use of particle-packed columns in most of the cases. Recently, siderophores-inspired synthetic analogs were used by Pallares *et al.* to separate lanthanide and actinides where an active bidentate hydroxypyridinonate group has been utilized for the complexation of lanthanides and actinide ions.^48^ Similarly, biopolymers, small-molecule lixiviants, peptides, and proteins are also utilized and studied to extract f-block elements.^3^

Keeping these in mind, the current work reports on the development of RP-HPLC methods for the selective separation of UO_2_^2+^ and Th^4+^ using dynamically modified C_18_ silica monolith columns embedded with four amide derivatives of *N*^1^,*N*^1^,*N*^3^,*N*^3^,*N*^5^,*N*^5^-hexa(alkyl = butyl/hexyl/octyl/decyl) benzene 1,3,5-tricarboxamide, denoted as HBBTA, HHBTA, HOBTA, and HDBTA, amidst more significant concentrations of lanthanide ions. The work explicitly describes the separation of UO_2_^2+^ and Th^4+^ ions, which are difficult to separate due to their similarity in intrinsic chemical properties. The usage of these analytes in industrial, nuclear and medicinal fields necessitates their isolation. It is well-known that amide-based chemo-markers are excellent extractants for actinides. In this work, the prudent choice and tuning of hydrophobic alkyl chains in the triamide ionophores display varying levels of interaction behavior with the C_18_ moiety through hydrophobic interaction to ensure uniform coating. The alkyl chain also helps enhance the ligand's non-leaching property since the proposed mobile phase is aqueous. The proposed method can be expressed/labeled as one of the most cost-effective LC-based techniques with reusable nature. The used modifiers can be removed entirely/stripped off after analysis, regenerating the bare column with 100% efficiency. Also, the novel ligand/modifier used in the current method proffers excellent future probabilities in designing ligands of similar structural formulations for extracting actinide ions from various sample matrices. The first of its use of an aromatic amphiphilic triamide-modified silica monolithic column having a reusability nature for the separation of UO_2_^2+^/Th^4+^ is the state-of-the-art nature of the proposed technique. This technique significantly advances the ease and efficiency of separating radionuclides of specific interest. The retention and elution patterns of UO_2_^2+^ and Th^4+^ ions using the triamide-based column modifiers have been studied using α-HIBA as the mobile phase and arsenazo(iii) as the post-column reagent (PCR). A detailed investigation has been carried out to study the influence of various analytical parameters, such as the role of mobile phase pH, amide concentration, mobile phase flow rate, *etc.*, on chromatographic performance. We have compared the effect of alkyl chain length in separation patterns to optimize the quantitative and qualitative separation of the target analytes. It should also be mentioned that the column modifier can be eluted out after use, retaining the bare monolith column without compromising its performance.

## 2. Materials and methods

### 2.1. Instrumentation and materials

A high-pressure liquid chromatographic system (JASCO LC 4000 Plus) consisting of dual solvent delivery pumps (JASCO PU 4180), a sampling valve fitted with a 20 μL loop (Rheodyne 7725, manual injector), PCR delivery pump (JASCO PU 4180) and a UV-vis spectrophotometer (JASCO UV 4070) was utilized. A reversed-phase C_18_ silica monolith column (Chromolith RP-18e, 100 × 4.6 mm), surface modified by dynamically coating with a series of lab-synthesized aromatic triamide derivatives, was used as the stationary phase. Arsenazo(iii) (0.15 mM, 1.5 mL min^−1^) was used as the PCR for the post-column derivatization reaction with the eluted UO_2_^2+^/Th^4+^ ions in a custom-designed T-connector loop. Finally, UO_2_^2+^/Th^4+^–arsenazo(iii) complexes were detected/quantified at 665 nm (*λ*_ma*x*_) using a UV-vis spectrophotometric detector. ChromNAV 4000 series software was employed to process the signal responses from the detector to obtain the chromatograms through a JASCO LC NET-II/ADC interface. A digital pH meter (Metrohm 913) was used to adjust the mobile phase pH, and the pH adjustments were conducted using dilute HCl/NaOH. A UV-vis spectrophotometer (Jasco V-670) was utilized to determine the amounts of triamides that were adsorbed onto the chromatographic column after the elution process. The prepared column modifiers are characterized using ^1^H & ^13^C NMR (Bruker 400 MHz, Advance model), FTIR spectrophotometer (Shimadzu Affinity 1 Spectrum model), and a CHN analyzer (Elemental VarioMicro Select EL model). The stock solutions of Ln^3+^ (Aldrich, 99.9%), UO_2_^2+^ (SRL, 96%), and Th^4+^ (SDFCL, 99%) were prepared from their respective nitrate salts. The α-HIBA (Sigma-Aldrich, >99% purity), metal ion stocks, and arsenazo(iii) (TCI, > 95%) solutions were prepared using ultrapure Milli-Q water of high purity and filtered (membrane filters, 0.22 μm, Merck) and degassed before usage. For the synthesis of the column modifiers, benzene-1,3,5-tricarbonyltrichloride, dibutyl amine, dihexyl amine, dioctyl amine, didecyl amine (Sigma-Aldrich, 98%), Triethylamine (Finar, 99%), dry diethyl ether (SD-fine, AR grade) were used.

### 2.2. Synthesis and characterization of the column modifiers

**Scheme 1:**
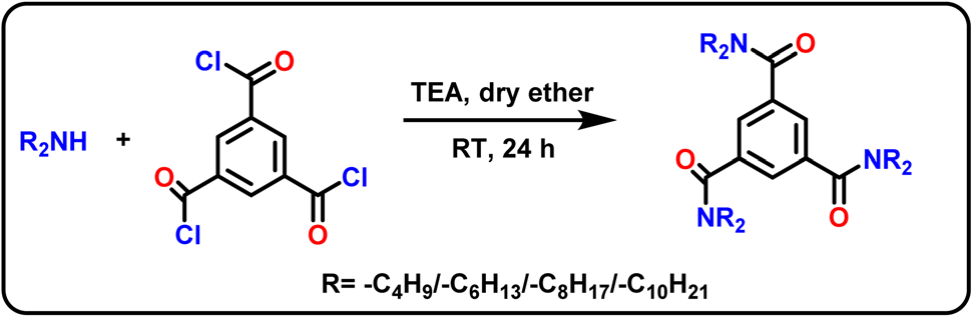
Synthesis of triamide column modifiers.

The column modifiers, *i.e.*, *N*^1^,*N*^1^,*N*^3^,*N*^3^,*N*^5^,*N*^5^-hexa(butyl/hexyl/octyl/decyl)benzene-1,3,5-tricarboxamide derivatives (HBBTA/HHBTA/HOBTA/HDBTA) were synthesized using benzene-1,3,5-tricarbonyl trichloride and dibutyl/dihexyl/dioctyl/dodecyl amine through a simple one-pot synthetic route. For this, benzene-1,3,5-tricarbonyl trichloride (1.0 mmol) was added to dry ether (25 mL) along with triethylamine (3.5 mmol) and was allowed to stir for 10 min at 0 °C. Then a mixture of 3.2 mmol of dialkylamine in dry ether was added to the acid chloride mixture using a pressure equalizer. The reaction was allowed to stir at room temperature for 24 h. The crude reaction mixture was washed with water, extracted with diethyl ether, and vacuum distilled to obtain a yellow-colored oily liquid. The same procedure was followed to prepare all four amide derivatives (Scheme 1), and the products were characterized using ^1^H & ^13^C-NMR, FT-IR spectra, and CHN elemental analysis.

#### HBBTA (yield: 93%)

^1^H NMR (Fig. S1†) (400 MHz, CDCl_3_), *δ*: 0.73 (t, *J* = 60.2 Hz, 18H), 1.06–1.30 (m, 12H), 1.4–1.55 (m, 12H), 3.13 (t, *J* = 30.70 Hz, 12H), 7.3 (s, 3H). ^13^C NMR (Fig. S2†) 400 MHz, CDCl_3_, *δ*: 168.94, 136.68, 124.58, 43.51, 28.53, 18.77, 12.68. IR frequencies (Fig. S3†): 2929 cm^−1^ (aromatic –C–H), 2872 cm^−1^ (aliphatic –C–H), 1627 cm^−1^ (–C=O), 1111 cm^−1^ (aliphatic –C–N). CHNS: %C 72.33 (72.88), %H 10.12 (10.56), %N 7.68 (7.73), %O 8.72 (8.83). The theoretical values are given in brackets.

### HHBTA (yield: 91%)

^1^H NMR (Fig. S4†) (400 MHz, CDCl_3_), *δ*: 0.86–0.90 (m, 18H), 1.20–1.22 (m, 36H), 1.48 (t, *J* = 55.60 Hz, 12H), 3.33 (t, *J* = 110.00 Hz, 12H), 7.37 (s, 3H). ^13^C NMR (Fig. S5†) 400 MHz, CDCl_3_, *δ*: 169.92, 137.70, 125.61, 44.80, 31.35, 28.77, 26.35, 22.49, 13.94. IR frequencies (Fig. S6†): 2926 cm^−1^ (aromatic –C–H), 2856 cm^−1^ (aliphatic –C–H), 1631 cm^−1^ (–C=O), 1112 cm^−1^ (aliphatic –C–N). CHNS: %C 75.48 (75.90), %H 11.21 (11.46), %N 5.30 (5.90), %O 6.59 (6.74).

### HOBTA (yield: 94%)

^1^H NMR (Fig. S7†) (400 MHz, CDCl_3_), *δ*: 0.79 (t, *J* = 7 Hz, 18H), 1.02–1.64 (m, 72H), 3.11–3.39 (m, 12H), 7.3 (s, 3H). ^13^C NMR (Fig. S8†) 400 MHz, CDCl_3_, *δ*: 169.41, 137.69, 125.62, 49.26, 44.82, 31.81, 29.70, 26.73, 14.09. IR frequencies (Fig. S9†): 2922 cm^−1^ (aromatic –C–H), 2852 cm^−1^ (aliphatic –C–H), 1635 cm^−1^ (–C=O), 1114 cm^−1^ (aliphatic –C–N). CHNS: %C 77.70 (77.76), %H 12.01 (12.02), %N 4.68 (4.77), %O 5.42 (5.45).

### HDBTA (yield: 89%)

^1^H NMR (Fig. S10†) (400 MHz, CDCl_3_), *δ*: 0.90 (t, *J* = 8 Hz, 18H), 1.1–1.64 (m, 96H), 3.19–3.47 (m, 12H), 7.38 (s, 3H). ^13^C NMR (Fig. S11†) 400 MHz, CDCl_3_, *δ*: 169.90, 137.69, 125.26, 44.84, 31.89, 29.56, 29.31, 22.68, 14.10. IR frequencies (Fig. S12†): 2926 cm^−1^ (aromatic –C–H), 2659 cm^−1^ (aliphatic –C–H), 1693 cm^−1^ (–C=O), 1107 cm^−1^ (aliphatic –C–N). CHN elemental analysis: %C 78.95 (79.02), %H 12.31 (12.40), %N 3.98 (4.01), %O 4.52 (4.58).

### 2.3. Dynamic column modification process

For the dynamic modification process, appropriate quantities of the secondary triamide derivatives (HBBTA/HHBTA/HOBTA/HDBTA) were weighed and dissolved separately in a methanol and water mixture. The stoichiometry of the MeOH : H_2_O ratio was optimized to ensure a composition that will not profoundly precipitate the amide ligands, as shown in Table S1 (ESI†). The coating solutions were thoroughly degassed and pumped through the C_18_ silica monolithic column at an optimum flow rate of 0.2 mL min^−1^ at ambient temperatures. Before coating, the column is pre-equilibrated with 50 mL of the same solvent mixture (MeOH: water). Moreover, the column was washed with water after modification and pre-equilibrated with 50 mL of mobile phase (0.1 M HIBA) to perform the metal ion separation studies. After the dynamic modification, the column should not be pumped with mobile phases consisting of organic solvents, which could elute the sorbed amide molecules. The column eluate during the modification process was collected to quantify the amide molecules that leave the column without getting adsorbed by a UV-visible spectrophotometer after extracting the same in diethyl ether. Finally, to accurately determine the amide modifier coated onto the C_18_ silica monolith column, the modified column was allowed to undergo a desorption process using 60 mL methanol as eluent at a flow rate of 0.2 mL min^−1^ after completing all the metal ion separation studies. The modifier-methanol solutions eluted from the column were collected and quantified (Table S1, ESI†).

### 2.4. Chromatographic procedure for the separation of UO_2_^2+^ & Th^4+^

The modified column was initially washed with water, followed by equilibration with the mobile phase (α-HIBA) of appropriate pH for 30 min at 1 mL min^−1^. A 20 μL of the analyte mixture is further injected into the modified column keeping the mobile phase flow rate at 1 mL min^−1^. The retentions of the analytes in the column were monitored by a UV-vis detector preceded by a post-column derivatization reaction using arsenazo(iii), where the PCR's flow rate was fixed at 1.5 mL min^−1^. However, the retention behaviors of the analytes on an unmodified column were performed under the same chromatographic conditions followed for the modified columns. The optimized conditions for the chromatographic separation of UO_2_^2+^ and Th^4+^ using the C_18_ silica monolithic columns that were modified with four triamide derivatives as given in Table S2 (ESI†). Reproducibility tests were conducted by recoating the column with the same ligand concentration after eluting the column modifiers from the column after analysis. For data reliability and reproducibility, tests were conducted *via* intra-day, inter-day, column-to-column, and batch-to-batch methods. All the obtained data were reproducible with an RSD ≤ 2.5%.

## 3. Results and discussions

### 3.1. Influence of pH of the mobile phase on the elution patterns of UO_2_^2+^ and Th^4+^

**Fig. 1:**
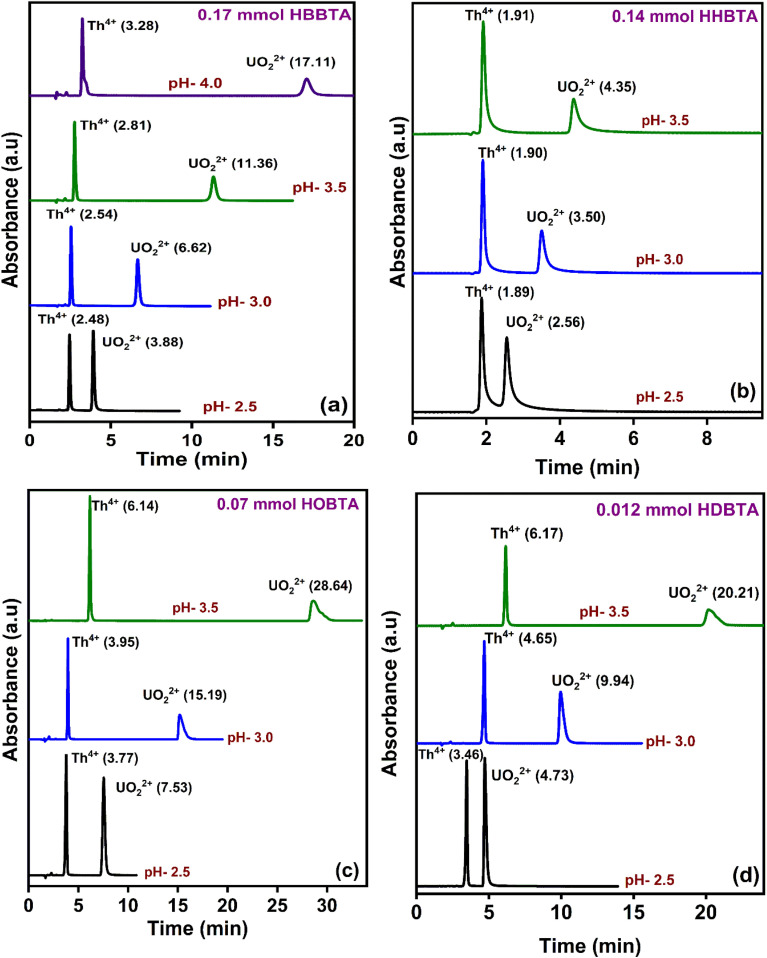
Retentions of UO_2_^2+^ and Th^4+^ in various HIBA pH (a) 0.17 mmol HBBTA, (b) 0.14 mmol of HHBTA, (c) 0.07 mmol of HOBTA and (d) 0.012 mmol of HDBTA modified monolith columns.

The elution order and the possible interaction pattern of UO_2_^2+^ and Th^4+^ with the modified columns were investigated using varying mobile phase pH, *i.e.*, 2.5–4.0 (Fig. 1). The chromatograms display an early elution for Th^4+^ ions compared to UO_2_^2+^, thus depicting the strong retention/complexation of UO_2_^2+^ with the column modifiers. In addition, it has also been observed that as the pH of the mobile phase increases, the retention time corresponding to both ions exhibits a gradual increase. Besides, all the triamide-based ligands show a similar pattern in separating UO_2_^2+^ and Th^4+^ ions due to their identical functional groups (amides), besides being neutral extractants. The chromatographic peak pattern of the HBBTA-modified column revealed well-resolved peaks for the sequential separation of Th^4+^ and UO_2_^2+^, at a mobile phase pH of 2.5, with a retention time of 2.48 and 3.88 min, respectively. A similar peak pattern was observed with the other amide-modified columns with a retention time of 1.89 and 2.56 min for HHBTA, 3.77 and 7.53 min for HOBTA, and 3.46 and 4.73 min for HDBTA for Th^4+^ and UO_2_^2+^, respectively. The observance of these separation patterns can be explained based on the preferential species formation of the UO_2_^2+^ and Th^4+^ ions with HIBA at various pH conditions, followed by their interactions with the triamide ligand derivatives surface adsorbed onto the C_18_ monolithic silica column. The existence of different [UO_2_^2+^/Th^4+^–IBA] complexes in the mobile phase (HIBA) medium as a function of pH variation is depicted below,
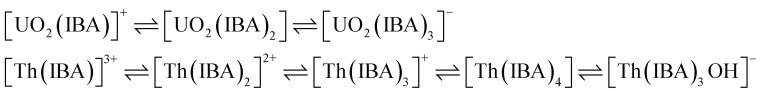


These species eventually interact with the hydrophobic amide moieties resulting in their separations. In the case of UO_2_^2+^, at lower pH, the cationic [UO_2_(IBA)]^+^ species predominates, whereas at higher pH, generally, the negatively charged anionic species [UO_2_(IBA)_3_]^−^ predominates in the solvent phase.^49^ Neutral [UO_2_(IBA)_2_] complexes are mainly observed as a predominant species at pH 3.0. It was found that a total of 58% of neutral + anionic IBA species of UO_2_^2+^ and Th^4+^ will be present at pH 3.0. However, at pH 3.5, this sum has been significantly enhanced to 83.5% and 92.1%, respectively. This means that beyond pH 3.0, the population of neutral [UO_2_(IBA)_2_] and anionic [UO_2_(IBA)_3_]^−^ inside the column increases. The high hydrophobicity due to the larger number of carbon moieties present in the neutral [UO_2_(IBA)_2_] and anionic [UO_2_(IBA)_3_]^−^ complexes results in a strong hydrophobic interaction between the [UO_2_(IBA)_3_]^−^ and the hydrophobic amide moieties forming a more stable amide interaction, thereby increasing the retention times.^50^ These [UO_2_(IBA)]^+^ cationic complexes have lesser interactions with the amide molecules in the stationary phase, thereby exhibiting shorter retention times for all four amide derivatives. However, Th^4+^ forms positively charged species predominantly at pH 2.5–3.0 and neutral/anionic complexes at pH 4.0 ([Th(IBA)_4_], [Th(IBA)_4_OH]^−^). This elucidates that the number of HIBA molecules surrounding thorium ions increases as the pH of the mobile phase increases, which directly contributes to the enhancement of hydrophobicity of the Th^4+^–IBA complexes, which increases the retention time in the stationary phase marginally. However, the lower retention of Th^4+^–IBA complexes despite having high hydrophobicity (due to the larger number of IBA moieties surrounding them) might be due to the possible hydrolysis generating [Th(IBA)_4_OH]^−^ complex. The overall formation constant of [Th(IBA)]^3+^, [Th(IBA)_2_]^2+^, [Th(IBA)_3_]^+^ & [Th(IBA)_4_] are 4.43, 8.15, 11.06, and 13.60, respectively. Similarly, for [UO_2_(IBA)]^+^, [UO_2_(IBA)_2_], [UO_2_(IBA)_3_]^−^ the values are 3.18, 5.13, and 6.67, respectively.^51^ Apart from the hydrophobic interaction, the complexation of the metal ions with the amide groups in the modifiers also plays a vital role in retaining UO_2_^2+^ and Th^4+^ complexes onto the column. Amides being neutral extractants, always prefers to form stable complexes with the neutral molecules of [UO_2_(IBA)_2_] and [Th(IBA)_4_] complexes and, in turn, contribute to increasing their retention times. Also, the unmodified C_18_ monolithic column did not resolve the UO_2_^2+^ and Th^4+^ ions under the same chromatographic conditions. The vital role played by the amide molecules on the separation factors by forming effective complexes in the stationary phase was thus exhibited by these separation profiles.

### 3.2. Retention behavior of UO_2_^2+^ and Th^4+^ as a function of the modifier concentrations

**Fig. 2:**
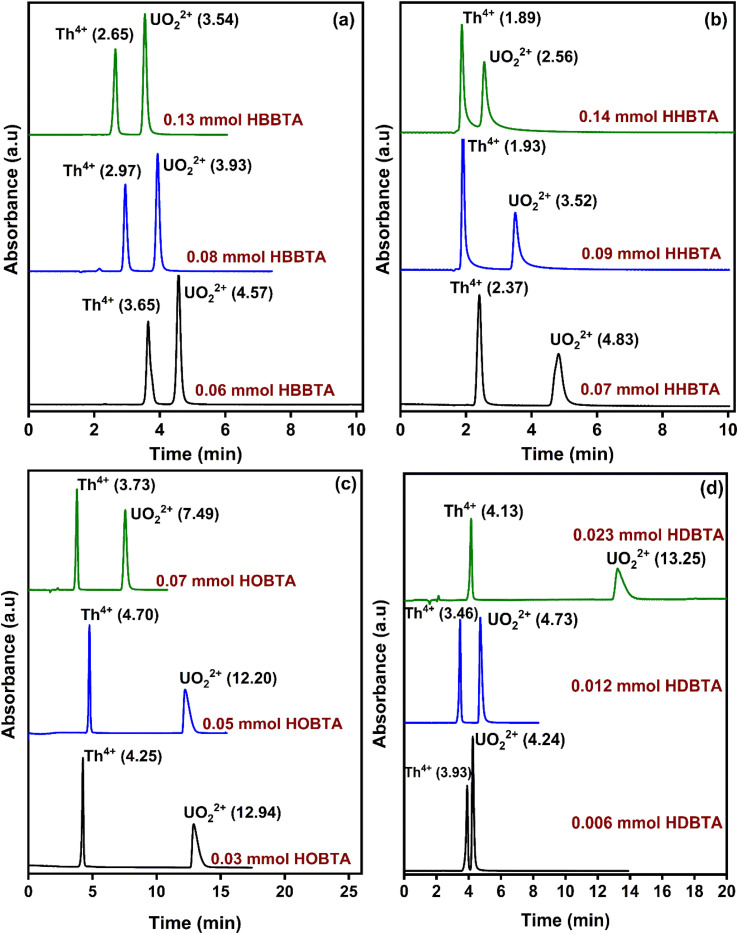
Retentions of UO_2_^2+^ and Th^4+^ as a function of various concentrations of column modifiers (a) HBBTA, (b) HHBTA, (c) HOBTA, and (d) HDBTA modified monolithic columns.

The influence of the amount of column modifiers on the resolution pattern of UO_2_^2+^ and Th^4+^ was investigated by experimenting with different concentrations of HBBTA/HHBTA/HOBTA/HDBTA modified stationary phases. A 20 μL of UO_2_^2+^ and Th^4+^ mixture (25 ppm each) was injected throughout the experiment. While experimenting with the role of modifier concentration on peak resolutions, the mobile phase and PCR flow rates were maintained constant at 1.0 mL min^−1^ and 1.5 mL min^−1^, respectively. Interestingly, a steady decrease in the retention times for Th^4+^ and UO_2_^2+^ was observed when the HBBTA content was increased from 0.06 mmol (3.65 min for Th^4+^ and 4.57 min for UO_2_^2+^) to 0.13 mmol (2.65 min for Th^4+^ and 3.54 min for UO_2_^2+^) (Fig. 2). Moreover, the same decreasing pattern was observed for the other two modified columns (HHBTA/HOBTA) on increasing the modifier content. More significant separation factors of the target analytes were achieved because of the predominant differences in the relative distributions of the analytes with the amide support and the HIBA mobile phase. At the same time, UO_2_^2+^ exhibited more outstanding retentions in the stationary phase than the Th^4+^ ions. But the observed decrease in the retention times with the increase in the amide-coated content was speculated to be due to the multi-layer amide coating, which reduces the accessibility of amide functional centers for the UO_2_^2+^ and Th^4+^ ions to form stable complexes. During the multi-layer coating, the hydrophobic carbon chains will be exposed more predominantly toward the surface, masking the amide groups meant for complexation. However, the role of amides on preferential retention of UO_2_^2+^ over Th^4+^ ions has been observed clearly with HDBTA-modified columns with a pattern showing increased retentions of analytes with increasing HDBTA amide content coating from 0.006 to 0.023 mmol on the C_18_ stationary phase. This further authenticates the dominant complexing property of UO_2_^2+^ ions with the amide groups in the HDBTA modifier, *i.e.*, the separation mechanism works predominantly based on the complexation process, nullifying the masking caused by the modifier moieties. The actinide ions are hard acids, and hard bases such as O have a greater affinity to form strong complexes with the metal ions. Amides are also considered as neutral extractants having significant electron-rich centers. These moieties are better candidates for extracting actinide ions through coordination with the amido oxygen and are proved to be robust and efficient agents in this complexation/extraction arena. However, some lagging must be addressed in separating these ions, especially amidst Lns of larger concentrations. As these ligands/modifiers exhibit similar behavior towards complexing Lns, the separate extraction of the selective actinide ions becomes less robust. We suggest a synergistic involvement of the use of LC-monolithic columns along with aromatic tri-amide modifiers, which can selectively separate the actinide ions under study within a 3 to 5 min time frame under optimized conditions. The lack of a sufficient modifier coated on the monolithic stationary phase ensures the non-separation behavior for lanthanide ions. In contrast, the amphiphilic aromatic tri-amide imparts wider accessibility of the functional groups projected in all directions for the metal ions to interact and complex. Also, the modifier's alkyl chains ensure strong amide moieties adhesion on the stationary support. From the chromatograms, the HBBTA ligand was identified as a promising candidate for the rapid resolution of UO_2_^2+^ and Th^4+^ within 5 min, wherein the unmodified bare C_18_ monolith column failed to resolve under similar chromatographic conditions. The order of elution, as well as the chromatogram pattern upon increasing the ligand content, was unaltered even if the hydrophobicity of the modifier ligand was varied. In the case of HOBTA and HDBTA modified supports (Fig. 2(c & d)), very low concentrations of the modifier could retain both Th^4+^ and UO_2_^2+^ significantly compared to the other investigated triamide derivatives. Also, as the alkyl chain length increases, the electron density on the C=O group of the amide functionality increases due to the inductive effect offered by the alkyl chains of the –NR_2_ units, which in turn increases the retention times of UO_2_^2+^ and Th^4+^. Hence, these modifiers will also be better alternatives for the bulk extraction of UO_2_^2+^ ions.

### 3.3. Influence of mobile phase flow rates on retention profiles

**Fig. 3:**
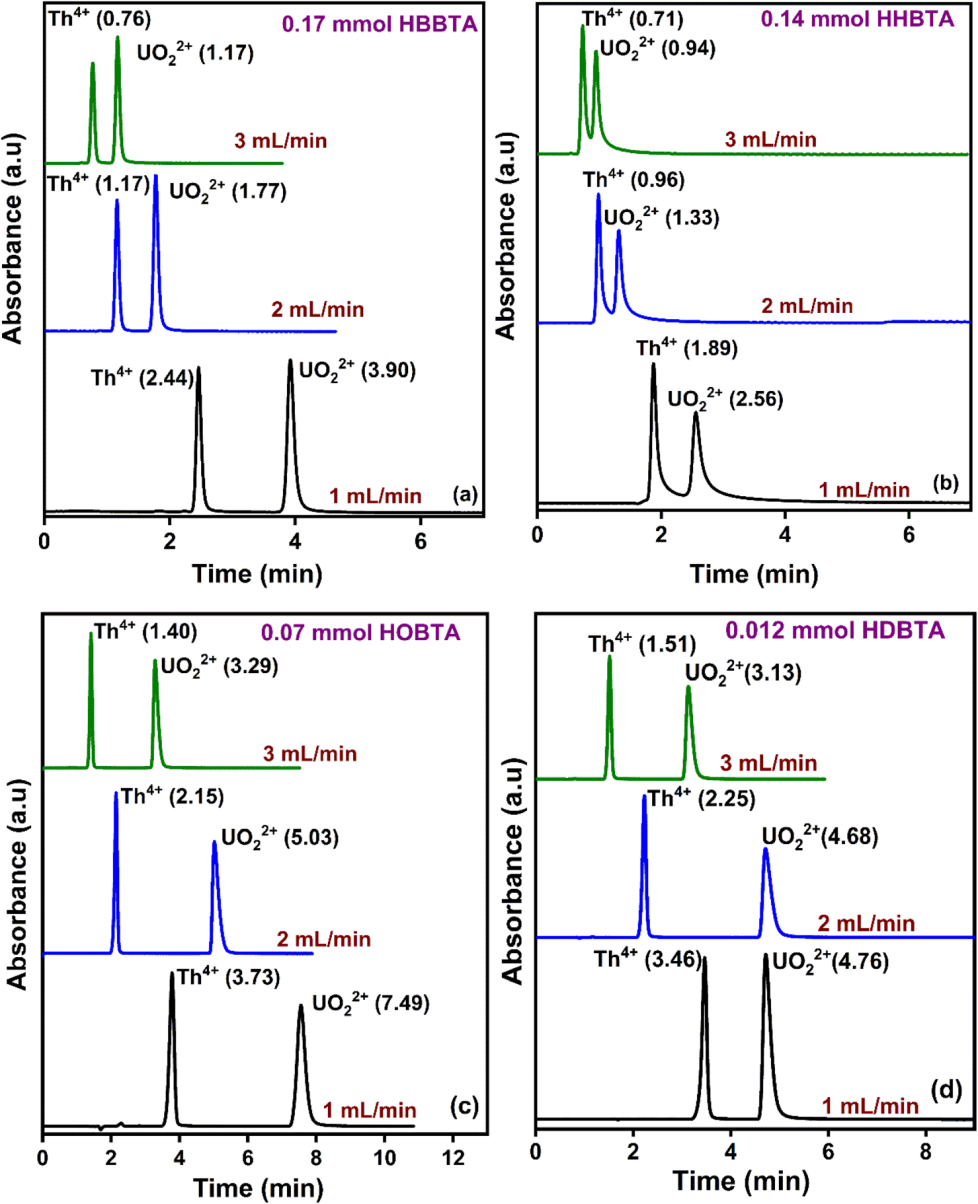
Retentions of UO_2_^2+^ and Th^4+^ with varying mobile phase flow rates (a) 0.17 mmol HBBTA column, (b) 0.14 mmol HHBTA column, (c) 0.07 mmol HOBTA column, and (d) 0.012 mmol HDBTA column.

The effect of eluent flow rates on the retention profiles of UO_2_^2+^ and Th^4+^ was studied by varying the mobile phase flow rates from 1 to 3 mL min^−1^ at pH 2.5 with different modified supports (Fig. 3). With all the amide derivatives, the retention times of the UO_2_^2+^ from Th^4+^ ions decrease with increasing flow rates. However, a well-resolved peak profile was observed at all the studied flow rates. At 3 mL min^−1^, rapid separation of UO_2_^2+^ and Th^4+^ ions was observed, with Th^4+^ eluting out of the HBBTA-modified column in just 0.76 min and UO_2_^2+^ in 1.17 min (Fig. 3a). All the other three ligand-modified columns exhibit similar results, as shown in Fig. 3(b–d).

### 3.4. Comparison of retention profiles of UO_2_^2+^ and Th^4+^ on different triamide derivatives

**Fig. 4:**
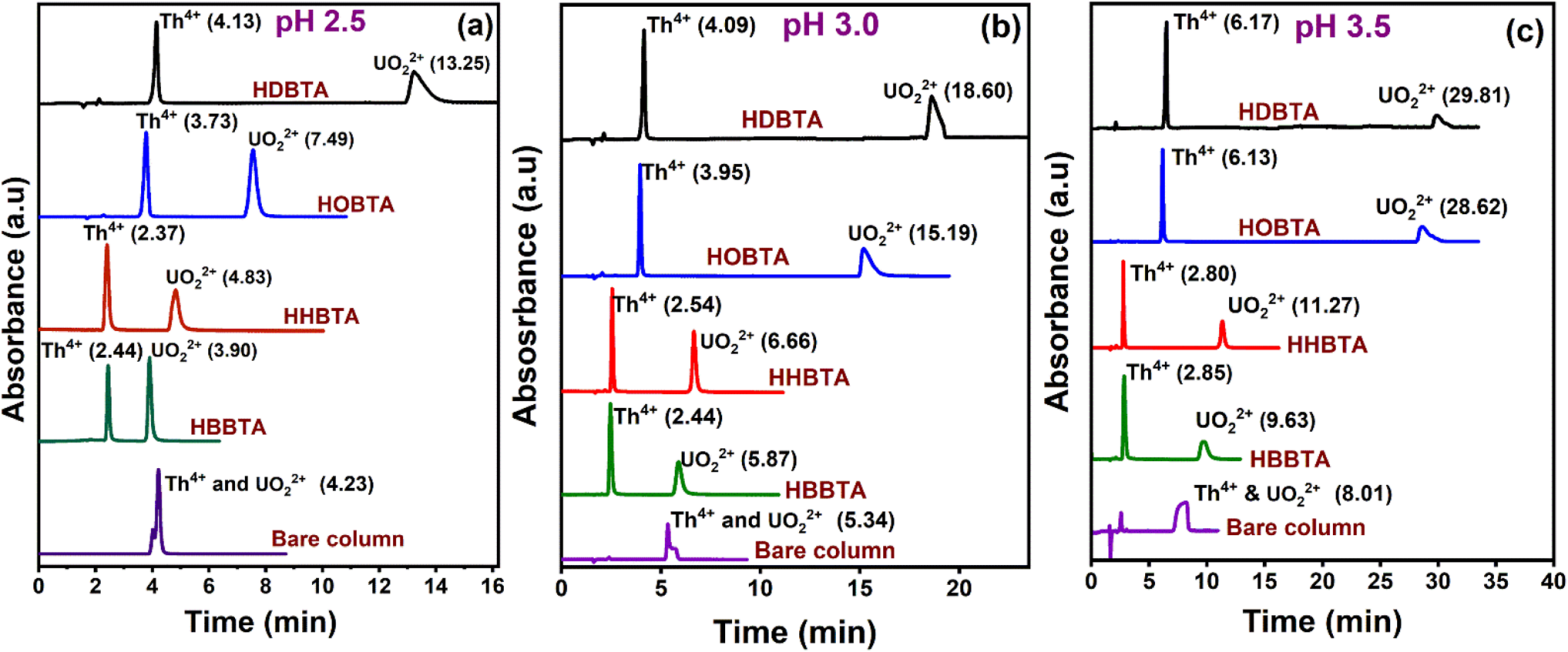
Retentions of UO_2_^2+^ and Th^4+^ with optimized HBBTA (0.17 mmol), HHBTA (0.07 mmol), HOBTA (0.07 mmol), and HDBTA (0.023 mmol) modified monolith column at mobile phase pH (a) 2.5, (b) 3.0 and (c) 3.5.

The comparison profiling attributed to the four modifiers has been studied by collating the retention profiles of UO_2_^2+^ and Th^4+^ mixture on 0.17 mmol HBBTA, 0.07 mmol HHBTA/HOBTA, and 0.023 mmol HDBTA modified columns at mobile phase pH of 2.5 (Fig. 4a), 3.0 (Fig. 4b), and 3.5 (Fig. 4c). The chromatograms distinguish the effect of variation in the alkyl chain constituent in the ligand, which directly affects the retention time of the analyte ions as one of the significant factors determining the separation is the hydrophobic interaction between the modified column and the analyte–IBA complex. From the chromatograms, a coherent transition/shift in the retention time of UO_2_^2+^ was noticed at each studied pH as the carbon chain length in the ligand increased. This can be explained as the additional hydrophobic interaction between the UO_2_^2+^–IBA complex with the modifier's long chain and the strong complexation between the analyte and the amide group in the ligand leading to higher retention of the analyte on the modified column/stationary phase. For example, the retention profile of UO_2_^2+^ at pH 2.5 on the four modified stationary phases imparts a gradual increase in the retention time as the carbon chain length increases from butyl to hexyl, then to octyl, and finally to decyl. An interesting observation regarding HDBTA is that, even at a concentration as low as 0.023 mmol, the high degree of hydrophobicity and complexing ability forces it to hold the UO_2_^2+^–IBA complex more, which results in a *t*_R_ of 13.25 min (at pH 2.5) which was exceptionally large compared to the other amide derivatives. With C_10_ decyl chains, the hydrophobicity of the ligands increases significantly, which in turn contributes to strong extraction of the actinide ions even with smaller ligand dosages. The effect of alkyl chain length in the modifier moiety on the retention of UO_2_^2+^ and Th^4+^ matters as it possesses a dual role in the proposed method. The primary role is a strong hydrophobic entity that ensures effective coating on the C_18_ silica monolithic support through which the leaching of the physically immobilized ligand/modifier on the stationary support was avoided. This way, more modifier moieties can be firmly fixed on the stationary support, which could eventually interact with the actinide ion under study. However, it is observed that the number of amide ligands which can be coated on the solid support also depends on the number of C_18_ moieties in the stationary phase. Hence, even though the strength of the coating and non-leachability property of the ligand on the stationary system can be enhanced by increasing the alkyl chain length, the number of effectively coated ligands will not be altered much on a given C_18_ system. One major problem associated with the increased number of carbon chains in the modifier is its difficulty preparing the coating solutions. As the ligand's hydrophobic nature increases, its precipitation on the coating solution on adding smaller aliquots of water increases tremendously. Also, on longer carbon chain lengths, the coiling effect will be predominant, which significantly reduces the efficacy of the coated column on separation. The role of alkyl chain length on diglycolamides (TODGA) derivative has been studied by Yadav *et al.*, where the results also corroborate with the observation in the present study. In that case, the loading capacity decreases as the alkyl chain length increases in the aliphatic modifier.^52^ Also, the butyl chains in the HBBTA ligand provide better accessibility for the actinide species around the carbonyl groups for better complexation (lesser steric hindrance for complexation).^53^ The second most important property associated with the alkyl groups lies in the separation/retention profiling of the target metal ions. The carbon chains enormously contribute to defining the retention time of the analytes as they induce the +I effect, demonstrating the electron density on the amido oxygen meant for complexing with the metal ions. As the carbon chain length increases, the +I effect will increase, which induces electron donation towards amido oxygen, enriching the complexing center and imparting a more basic character to the molecular structure. The high electron-donating capability of decyl chains compared to octyl, hexyl, and butyl, where the electron-donating capacity follows the order decyl > octyl > hexyl > butyl in the ligand enriches the electron density around the amido oxygen, forming stable complexes with UO_2_^2+^ and Th^4+^, thereby increasing the retention times. This way, stronger retention of the analytes was expected in higher alkyl groups. For example, even with a concentration as low as 0.023 mmol of HDBTA, a retention time of 13.25 min was observed for UO_2_^2+^. Besides, the longer alkyl chain groups will impart a severe steric hindrance, reducing the accessibility of the ligands for the analyte ions to complex. However, considering all the parameters/influences of the carbon chain in the modifier, a higher concentration coating of HDBTA ligand was not achieved due to their practical incapability of preparing coating solutions, owing to their high level of hydrophobic nature.

One another interesting observation was that Th^4+^ showed/exhibited only a marginal difference in its *t*_R_ value because of the lesser hydrophobicity associated with the cationic Th^4+^–IBA complex at pH 2.5 and the weak complexing ability with the amide groups compared with that of UO_2_^2+^. However, as the hydrophobicity of the ligand/modifier increases, the retention time for Th^4+^ is also increasing, which is evident from the shift in the observed *t*_R_ values as the carbon chain length increases to octyl and decyl. Interestingly, all four modified columns at all the studied three mobile phase pH conditions resolved both UO_2_^2+^ and Th^4+^ with excellent peak shape, as the bare C_18_ column (unmodified) failed to resolve under similar conditions.

### 3.5. Selective retention of UO_2_^2+^ and Th^4+^ ions over lanthanides

**Fig. 5:**
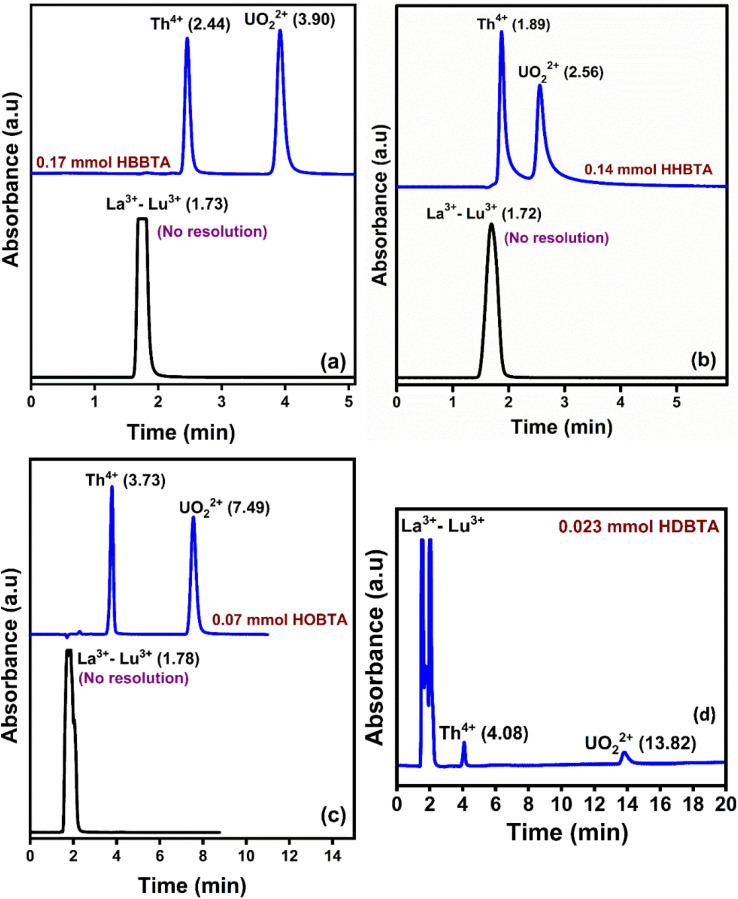
Retention of Ln^3+^, UO_2_^2+^, and Th^4+^ on (a) 0.17 mmol HBBTA, (b) 0.14 mmol HHBTA, and (c) 0.07 mmol HOBTA modified monolithic column. (d) Retention of Ln^3+^, UO_2_^2+^, and Th^4+^ on 0.023 mmol HDBTA modified monolithic column.

To ascertain the selective separation of UO_2_^2+^ and Th^4+^ amidst Lns, a 20 μL of a mixture containing UO_2_^2+^ (25 ppm), Th^4+^ (25 ppm), and lanthanoids (La^3+^–Lu^3+^, except Pm^3+^ 50 ppm each) was injected onto a 0.023 mmol HDBTA modified column. The Lns are frequently encountered matrix constituents in various UO_2_^2+^ and Th^4+^ sources, and the chromatogram is depicted in Fig. 5(d). The chromatogram showed that the injected mixture of 14 lanthanoids came out incredibly early (<2.0 min) as an unresolved peak showcasing its non-interaction with the triamide ligand-modified stationary phase. These findings show that the novel triamide ligands modified columns can be utilized exclusively to selectively separate and isolate UO_2_^2+^ and Th^4+^ ions from complex matrices. The poor retention behaviors of lanthanoids can be justified by the lesser number of amide moieties and C_18_ moieties in the monolith column, which was insufficient for lanthanoids to interact strongly with the column modifiers to form a stable complex. The individual retention behavior of the lanthanides and actinides on the three modified columns is shown in Fig. 5(a–c), which depicted a similar retention behavior to that of the HDBTA modified column.

## 4. Concluding remarks

Herein we report an easy, facile, and effective method for the systematic and selective separation of UO_2_^2+^ and Th^4+^ by dynamically modifying reversed-phase C_18_ silica monolithic columns using four novel hydrophobic aromatic triamide ionophoric ligands. The reproducibility and reusability have been evaluated through run-to-run, day-to-day, batch-to-batch, and column-to-column analysis. The work also compares the effect of amide hydrophobicity on the separation profile of UO_2_^2+^ and Th^4+^ by varying the alkyl chain length (ranging from butyl to decyl) in the synthesized triamide ligand molecules. The results revealed that with 0.023 mM HDBTA modified column, the separation of UO_2_^2+^/Th^4+^ was achieved at 13.25 and 4.13 min, respectively. Also, the studies conclude that the optimum coating concentration of HBBTA, HHBTA and HOBTA were 0.17 mM, 0.07 mM and 0.07 mM, respectively. The coated amide content formed stable complexes with [UO_2_^2+^/Th^4+^–IBA] species, resulting in good peak resolution, which was impossible with unmodified C_18_ silica monolithic columns. The method can be further extended for scale-up measurements using preparative columns where the aromatic triamide derivatives can separate and extract the target analytes. The modifier can be intuitively tuned for other relative applications such as sensing, solid-phase extraction, preconcentration, *etc.* Also, the method shows superior properties in separating UO_2_^2+^ and Th^4+^ amidst all lanthanoids, even if present in multi-fold concentrations.

## Data availability statement

Data is available on request from the authors.

## Author contributions

Aswanidevi, Thirumalai and Suchashrita Mitra: formal analysis, investigation, methodology, validation, writing – original draft. Brahmananda Rao, Sivaraman and Pitchaiah: conceptualization, methodology, validation, funding acquisition. Prabhakaran and Akhila Maheswari: visualization, conceptualization, investigation, methodology, software, supervision, validation, writing – review & editing, funding acquisition, project administration.

## Conflicts of interest

The authors declare that they have no known competing interests that could have appeared to influence the work reported in this paper.

## Supplementary Material

RA-013-D2RA07495H-s001

## References

[cit1] Hudson M. J., Harwood L. M., Laventine D. M., Lewis F. W. (2013). Inorg. Chem..

[cit2] Jaison P. G., Telmore V. M., Kumar P., Aggarwal S. K. (2011). J. Chromatogr. Sci..

[cit3] Mattocks J. A., Cotruvo J. A. (2020). Chem. Soc. Rev..

[cit4] Jaison P. G., Telmore V. M., Kumar P., Aggarwal S. K. (2009). J. Chromatogr. A.

[cit5] Kumar P., Jaison P. G., Telmore V. M., Paul S., Aggarwal S. K. (2013). Int. J. Anal. Mass Spectrom. Chromatogr..

[cit6] Schwantes J. M., Rundberg R. S., Taylor W. A., Vieira D. J. (2006). J. Alloys Compd..

[cit7] Ansari S. A., Mohapatra P. K. (2017). J. Chromatogr. A.

[cit8] Hu Y., Drouin E., Larivière D., Kleitz F., Fontaine F. G. (2017). ACS Appl. Mater. Interfaces.

[cit9] Fisher A., Kara D. (2016). Anal. Chim. Acta.

[cit10] Bera S., Balasubramanian R., Datta A., Sajimol R., Nalini S., Lakshmi Narasimhan T. S., Antony M. P., Sivaraman N., Nagarajan K., Vasudeva Rao P. R. (2013). Int. J. Anal. Mass Spectrom. Chromatogr..

[cit11] Truscott J. B., Jones P., Fairman B. E., Hywel Evans E. (2001). J. Chromatogr. A.

[cit12] Dupuis E., Isnard H., Chartier F. (2022). J. Anal. At. Spectrom..

[cit13] Hang W., Zhu L., Zhong W., Mahan C. (2004). J. Anal. At. Spectrom..

[cit14] Schmidt H., Wilden A., Modolo G., Bosbach D., Santiago-Schübel B., Hupert M., Mincher B. J., Mezyk S. P., Švehla J., Grüner B., Ekberg C. (2021). Radiat. Phys. Chem..

[cit15] Wang Z., Pu N., Tian Y., Xu C., Wang F., Liu Y., Zhang L., Chen J., Ding S. (2019). Inorg. Chem..

[cit16] Sun T., Xu C., Fu J., Chen Q., Chen J., Shen X. (2017). Sep. Purif. Technol..

[cit17] Muller J. M., Galley S. S., Albrecht-Schmitt T. E., Nash K. L. (2016). Inorg. Chem..

[cit18] Kaur M., Zhang H., Martin L., Todd T., Qiang Y. (2013). Environ. Sci. Technol..

[cit19] Afsar A., Distler P., Harwood L. M., John J., Westwood J. (2017). Chem. Commun..

[cit20] Liu Y., Shao X., Bu W., Qin Z., Ni Y., Wu F., Yang C., Wang X. (2022). Chin. Chem. Lett..

[cit21] Martínez J., Baciu T., Peñalver A., Aguilar C., Borrull F. (2019). J. Environ. Radioact..

[cit22] Roane J. E., DeVol T. A., Leyba J. D., Fjeld R. A. (2003). J. Environ. Radioact..

[cit23] Jia G., Buchetti M., Conti D., Magro L., Mariani S. (2022). Appl. Radiat. Isot..

[cit24] Lindahl P., Olszewski G., Eriksson M. (2022). Appl. Radiat. Isot..

[cit25] Harrison J. J., Zawadzki A., Chisari R., Wong H. K. Y. (2011). J. Environ. Radioact..

[cit26] Darge A. W., Gera Y., DeVol T. A., Husson S. M. (2020). React. Funct. Polym..

[cit27] Fullmer W. K., Bliznyuk V. N., Seliman A. F., Powell B. A., Husson S. M., DeVol T. A. (2022). J. Environ. Radioact..

[cit28] Benedik L., Pilar A. M., Prosen H., Jaćimović R., Povinec P. P. (2021). Appl. Radiat. Isot..

[cit29] Cabrera K., Lubda D., Eggenweiler H. M., Minakuchi H., Nakanishi K. (2000). J. Sep. Sci..

[cit30] Ali I., Gaitonde V. D., Aboul-Enein H. Y. (2009). J. Chromatogr. Sci..

[cit31] Ikegami T., Tanaka N. (2004). Curr. Opin. Chem. Biol..

[cit32] Maheswari M. A., Subramanian M. S. (2004). Sep. Sci. Technol..

[cit33] Maheswari M. A., Subramanian M. S. (2005). Solvent Extr. Ion Exch..

[cit34] Husain M., Ansari S. A., Mohapatra P. K., Gupta R. K., Parmar V. S., Manchanda V. K. (2008). Desalination.

[cit35] Horwitz E. P., Chiarizia R., Dietz M. L., Diamond H., Nelson D. M. (1993). Anal. Chim. Acta.

[cit36] Chandrasekar A., Suresh A., Joshi M., Sundararajan M., Ghanty T. K., Sivaraman N. (2019). Sep. Purif. Technol..

[cit37] Grüner B., Plešek J., Báča J., Císařová I., Dozol J. F., Rouquette H., Viňas C., Selucký P., Rais J. (2002). New J. Chem..

[cit38] Annam S., Gopakumar G., Brahmmananda Rao C. V. S., Sivaraman N., Sivaramakrishna A., Vijayakrishna K. (2018). Inorg. Chim. Acta.

[cit39] Sivaraman N., Subramaniam S., Srinivasan T. G., Vasudeva Rao P. R. (2002). J. Radioanal. Nucl. Chem..

[cit40] Sivaraman N., Kumar R., Subramaniam S., Vasudeva Rao P. R. (2002). J. Radioanal. Nucl. Chem..

[cit41] Vidyalakshmi V., Subramanian M. S., Sivaraman N., Srinivasan T. G., Vasudeva Rao P. R. (2004). J. Liq. Chromatogr. Relat. Technol..

[cit42] Raju C. S. K., Subramanian M. S., Sivaraman N., Srinivasan T. G., Vasudeva Rao P. R. (2007). J. Chromatogr. A.

[cit43] Raut V. V., Roy S. P., Das M. K., Jeyakumar S., Ramakumar K. L. (2013). Int. J. Anal. Mass Spectrom. Chromatogr..

[cit44] Datta A., Sivaraman N., Srinivasan T. G., Vasudeva Rao P. R. (2010). Radiochim. Acta.

[cit45] Datta A., Sivaraman N., Srinivasan T. G., Vasudeva Rao P. R. (2011). Radiochim. Acta.

[cit46] Deepika P., Sivaraman N., Sabharwal K. N., Srinivasan T. G., Vasudeva Rao P. R. (2011). Radiochim. Acta.

[cit47] Raut N. M., Jaison P. G., Aggarwal S. K. (2004). J. Chromatogr. A.

[cit48] Pallares R. M., Hébert S., Sturzbecher-Hoehne M., Abergel R. J. (2021). New J. Chem..

[cit49] Akhila Maheswari M., Prabhakaran D., Subramanian M. S., Sivaraman N., Srinivasan T. G., Vasudeva Rao P. R. (2007). Talanta.

[cit50] Datta A., Sivaraman N., Viswanathan K. S., Ghosh S., Srinivasan T. G., Vasudeva Rao P. R. (2013). Radiochim. Acta.

[cit51] Smith R. M., Martell A. E. (1983). Biochem. Educ..

[cit52] Yadav A. G., Gujar R. B., Valsala T. P., Sathe D. B., Bhatt R. B., Mohapatra P. K. (2022). J. Chromatogr. A.

[cit53] Mowafy E. A., Aly H. F. (2007). Solvent Extr. Ion Exch..

