# Supplementary material for: Fast and selective reversed-phase high performance liquid chromatographic separation of UO22+ and Th4+ ions using surface modified C18 silica monolithic supports with target specific ionophoric ligands
Source: RSC Adv. 2023 Jan 23;13(5):3317–28. doi: 10.1039/d2ra07495h (PMC9869661; doi:10.1039/d2ra07495h)
Supplement: RA-013-D2RA07495H-s001 [file RA-013-D2RA07495H-s001.pdf]

Electronic Supplementary Material (ESI) for RSC Advances.
This journal is © The Royal Society of Chemistry 2023

Electronic Supporting Information

Fast and Selective Reversed-Phase High-Performance Liquid Chromatographic Separation of UO_2^{2+} and Th^{4+} Ions using Surface Modified C_{18} Silica Monolithic Supports with Target Specific Ionophoric Ligands

Aswanidevi Kongasseri [1], Thirumalai Madhesan [1], Suchashrita Mitra [1], C V S Brahmmananda Rao [2], Sivaraman Nagarajan [2], Pitchaiah Kancharlapalli Chinaraga [2], Prabhakaran Deivasigamani [1,*], Akhila Maheswari Mohan [1,*]

[1] *Department of Chemistry, School of Advanced Sciences, Vellore Institute of Technology (VIT), Vellore, Tamil Nadu 632014, India*

[2] *Fuel Chemistry Division, Materials Chemistry and Metal Fuel Cycle Group, Indira Gandhi Centre for Atomic Research (IGCAR), Kalpakkam, Tamil Nadu 603102, India*

Table of Contents

Fig. S1	^{1}H NMR spectrum of HBBTA
Fig. S2	^{13}C NMR spectrum of HBBTA
Fig. S3	FT-IR spectrum of HBBTA
Fig. S4	^{1}H NMR spectrum of HHBTA
Fig. S5	^{13}C NMR spectrum of HHBTA
Fig. S6	FT-IR spectrum of HHBTA
Fig. S7	^{1}H NMR spectrum of HOBTA
Fig. S8	^{13}C NMR spectrum of HOBTA
Fig. S9	FT-IR spectrum of HOBTA
Fig. S10	^{1}H NMR spectrum of HDBTA
Fig. S11	^{13}C NMR spectrum of HDBTA
Fig. S12	FT-IR spectrum of HDBTA
Table. S1	Exact amount of the modifiers adsorbed onto the monolithic support
Table. S2	Optimized chromatographic conditions

I. ^{1}H & ^{13}C NMR & FT-IR spectra of HBBTA

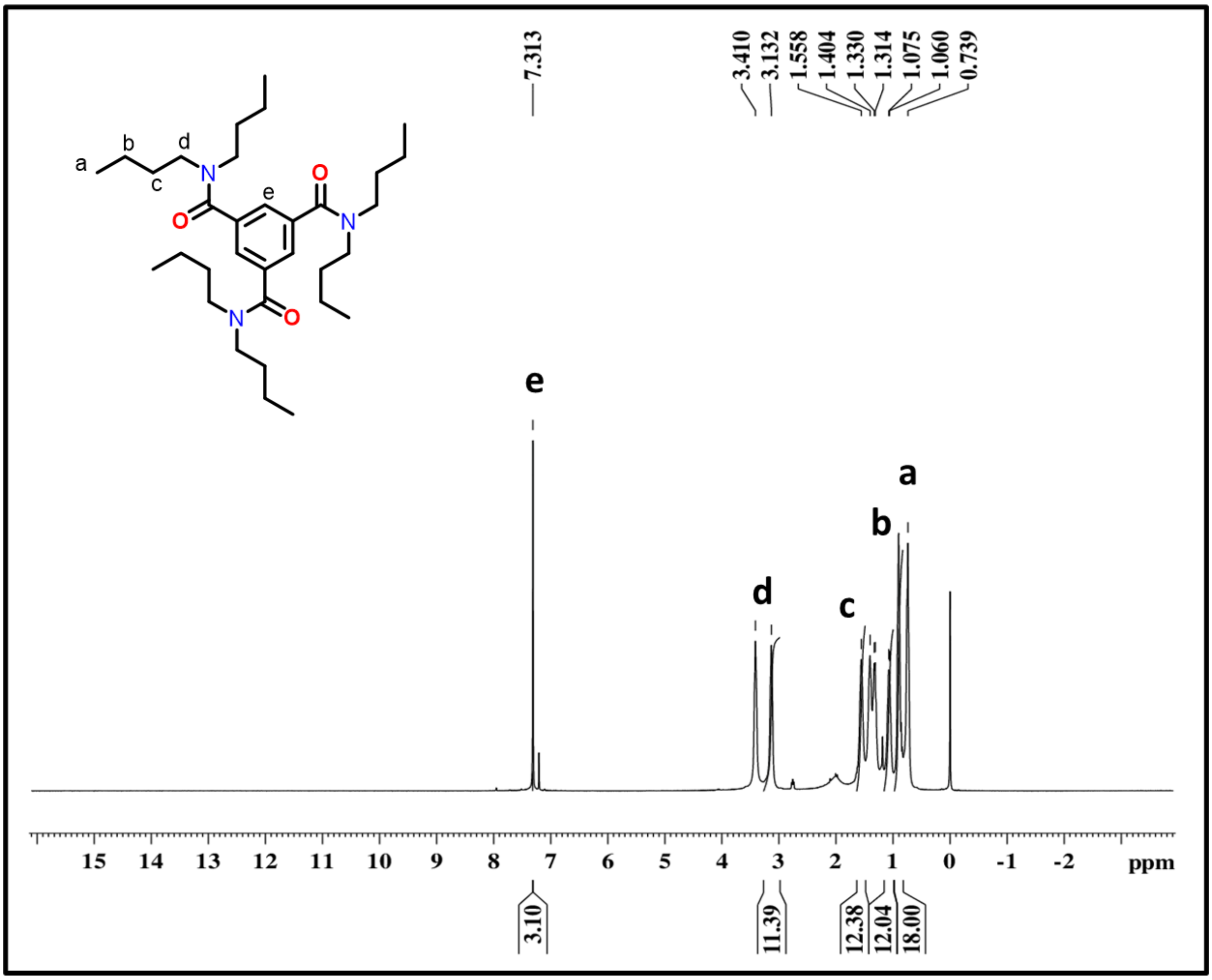

Fig. S1 ^{1}H NMR spectrum of HBBTA

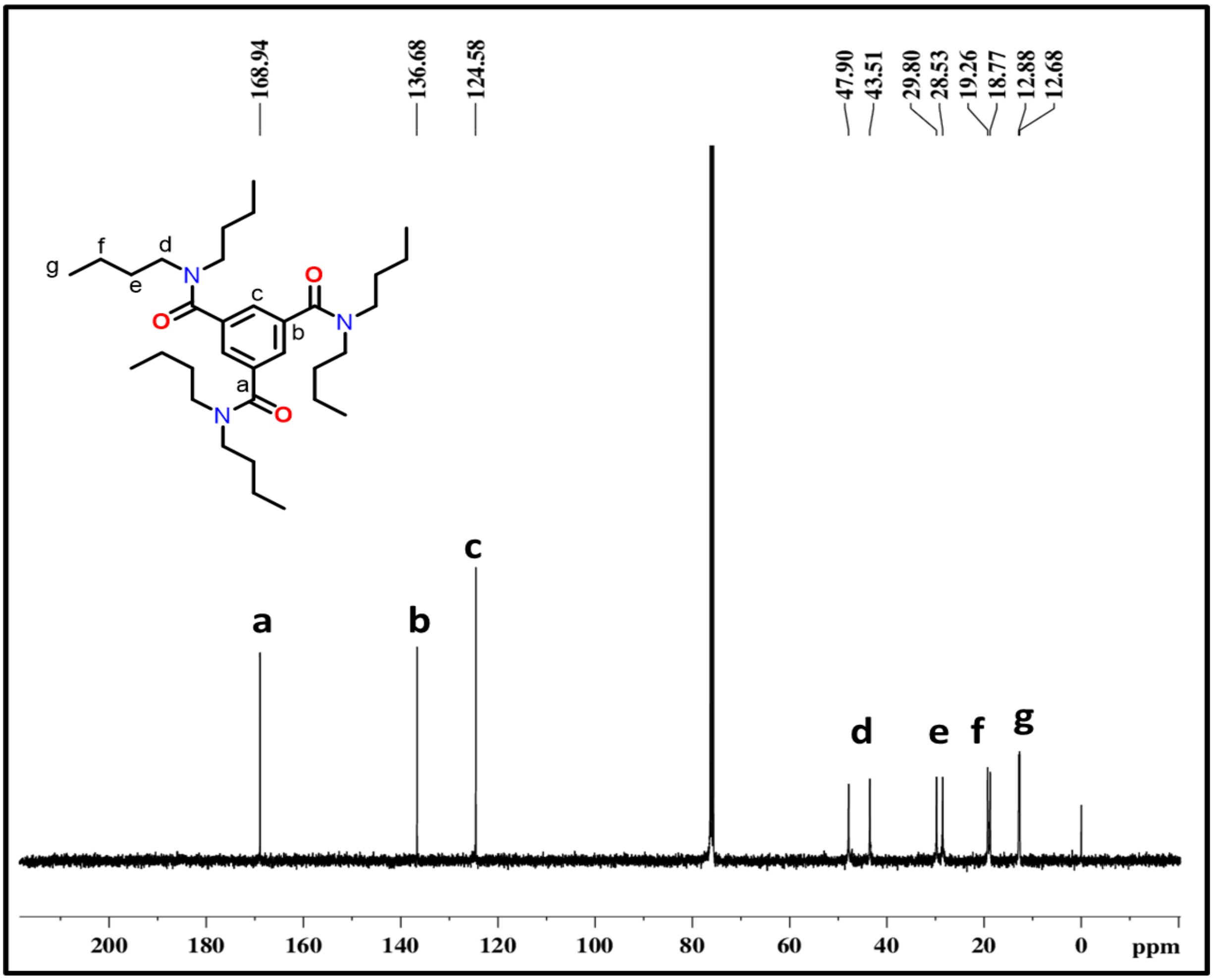

Fig. S2 ^{13}C NMR spectrum of HBBTA

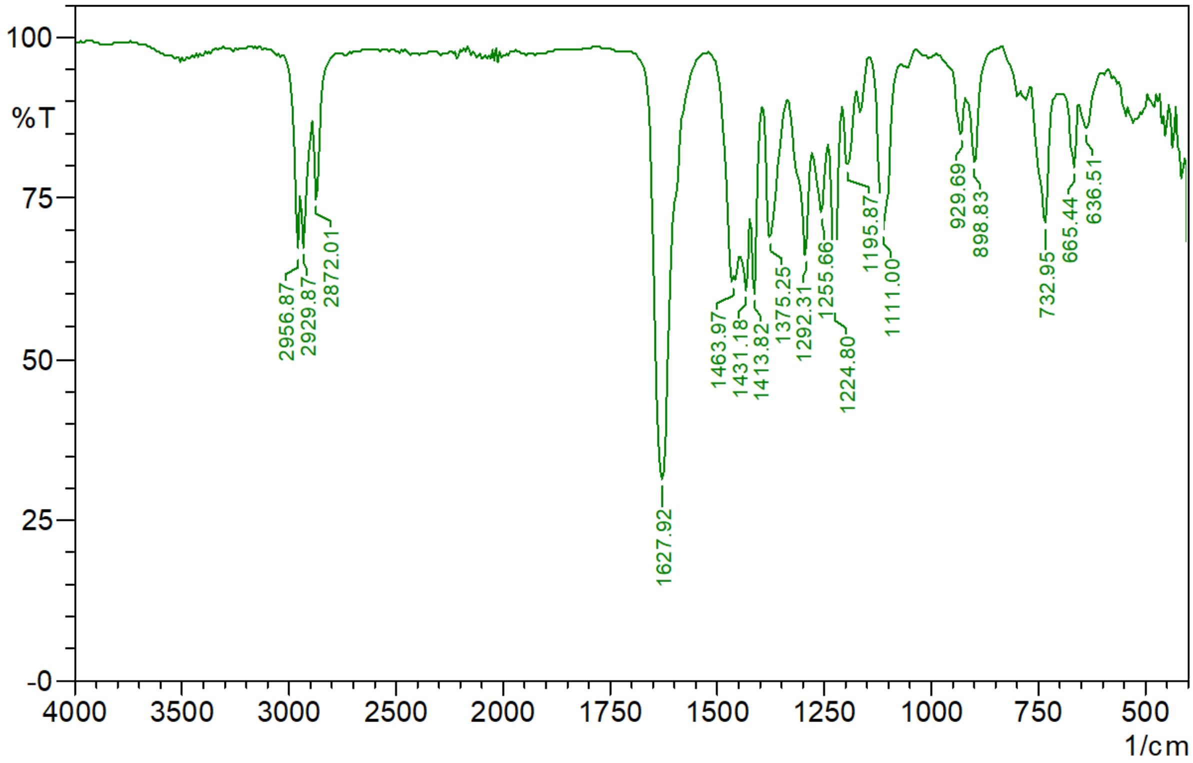

Fig. S3 FT-IR spectrum of HBBTA

II. ^{1}H & ^{13}C NMR & FT-IR spectra of HHBTA

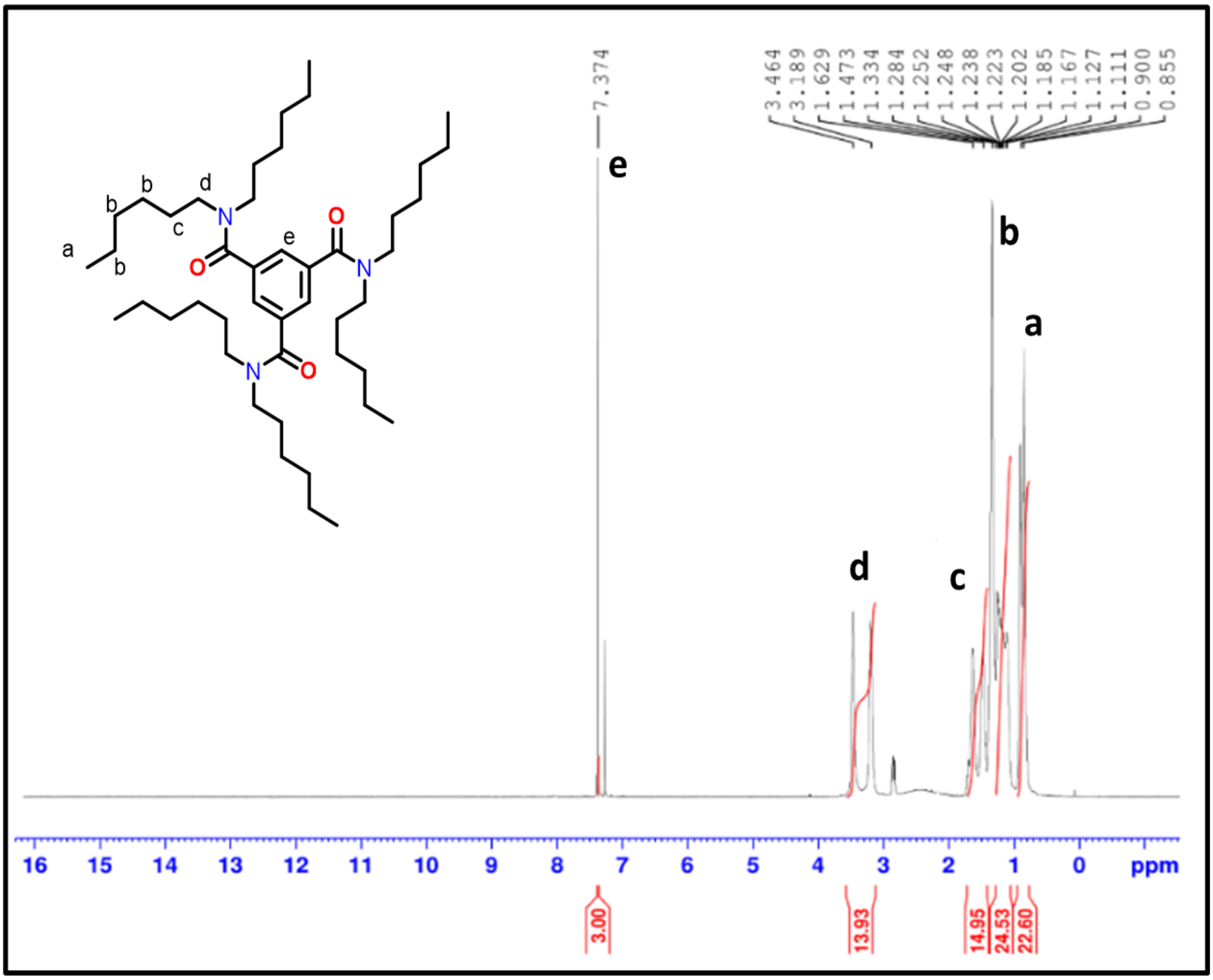

Fig. S4 ^{1}H NMR spectrum of HHBTA

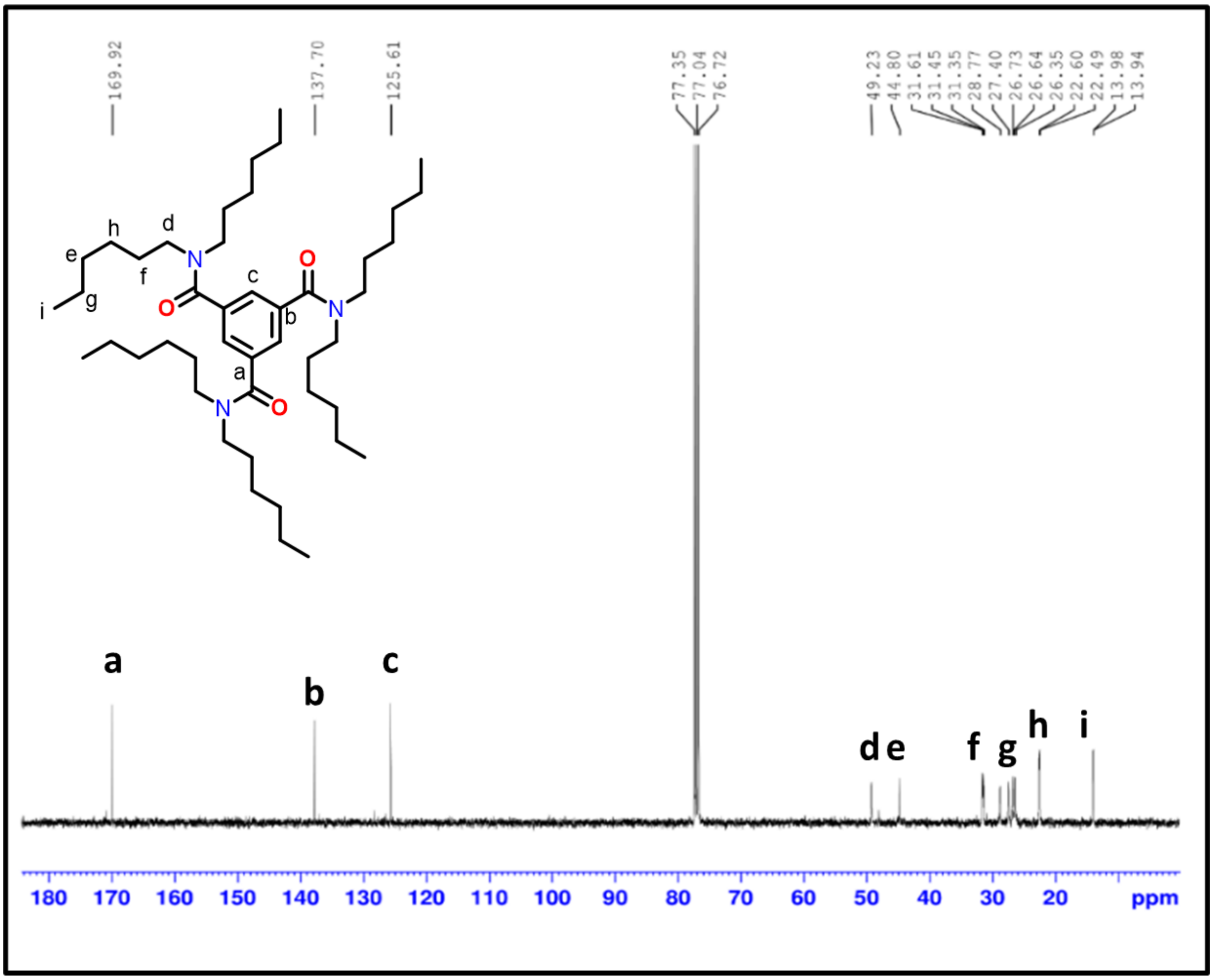

Fig. S5 ^{13}C NMR spectrum of HHBTA

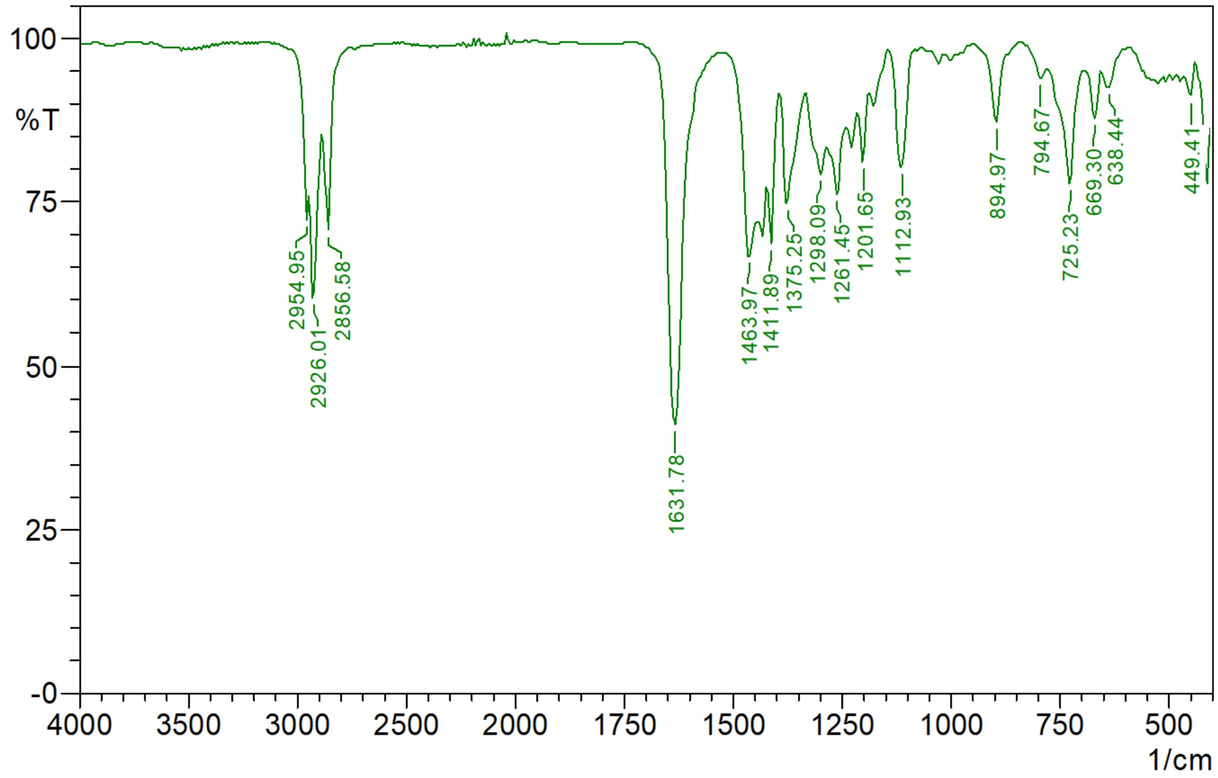

Fig. S6 FT-IR spectrum of HHBTA

III. ^{1}H & ^{13}C NMR & FT-IR spectra of HOBTA

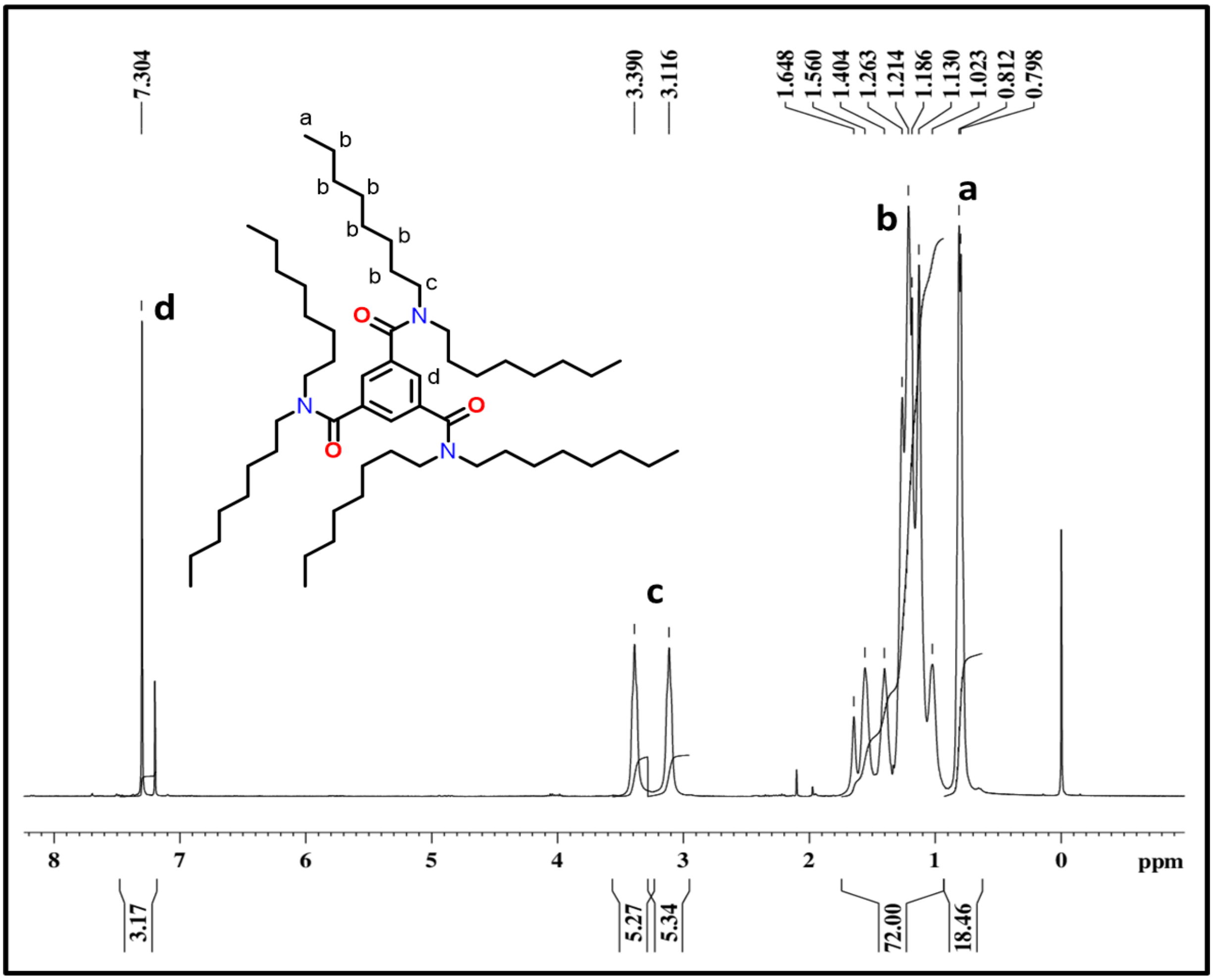

Fig. S7 ^{1}H NMR spectrum of HOBTA

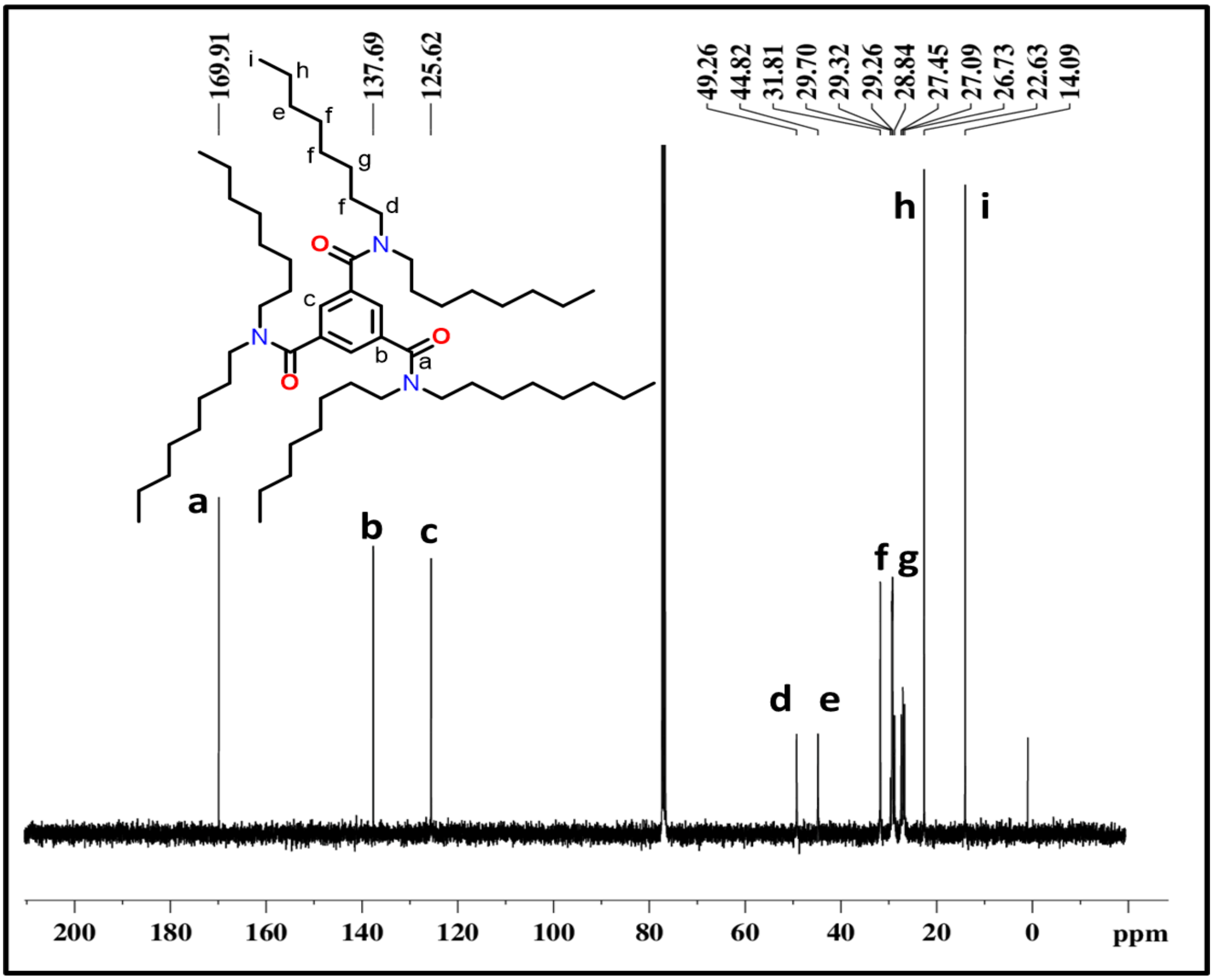

Fig. S8 ^{13}C NMR spectrum of HOBTA

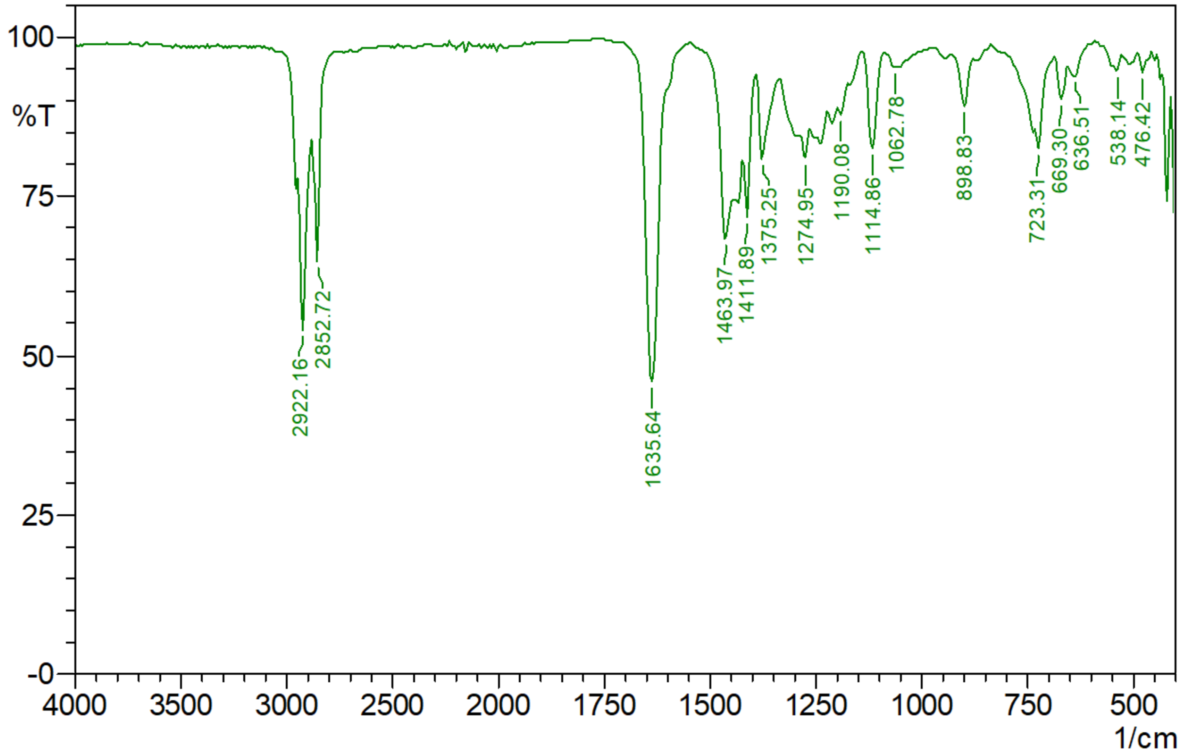

Fig. S9 FT-IR spectrum of HOBTA

IV. ^{1}H & ^{13}C NMR & FT-IR spectra of HDBTA

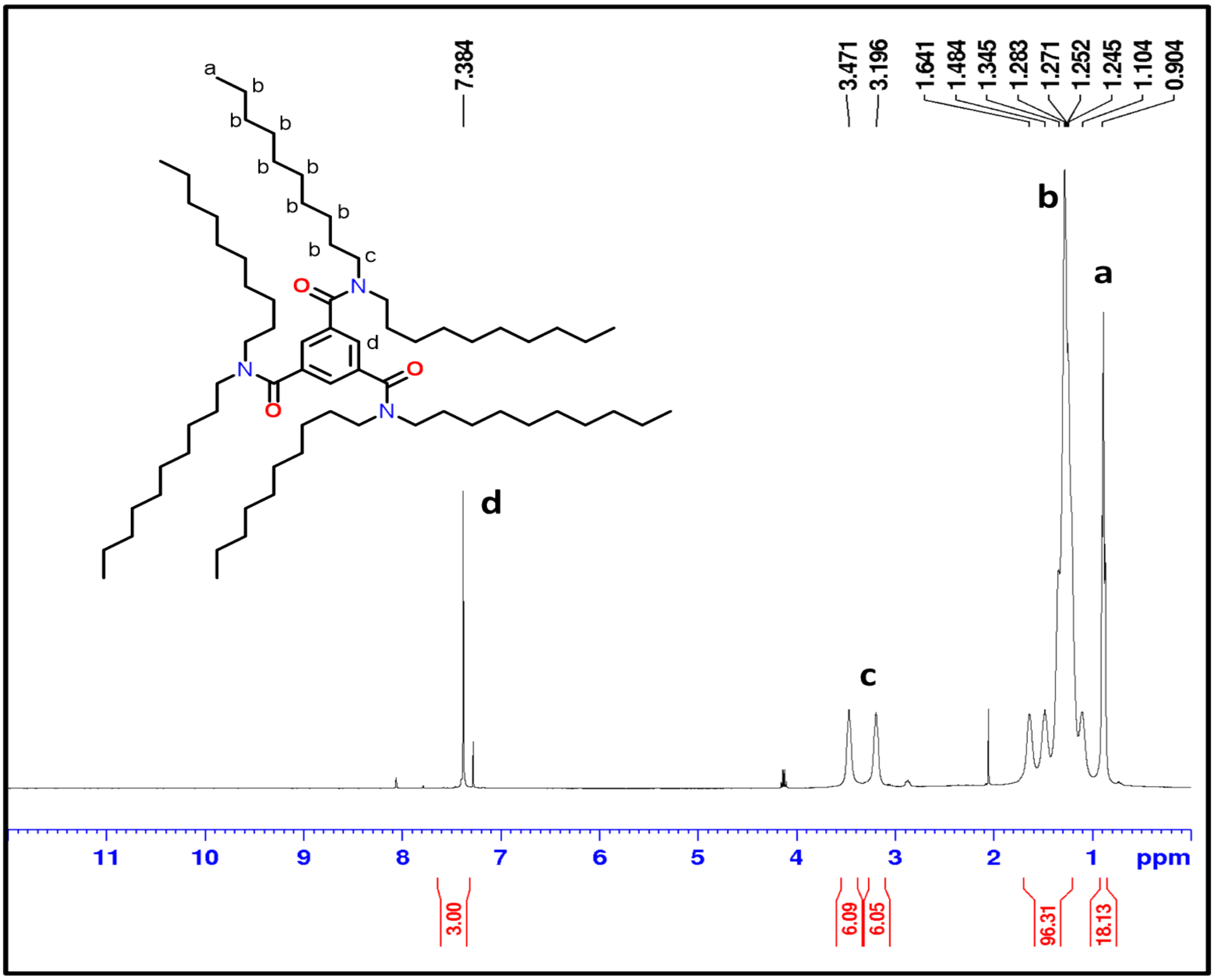

Fig. S10 ^{1}H NMR spectrum of HDBTA

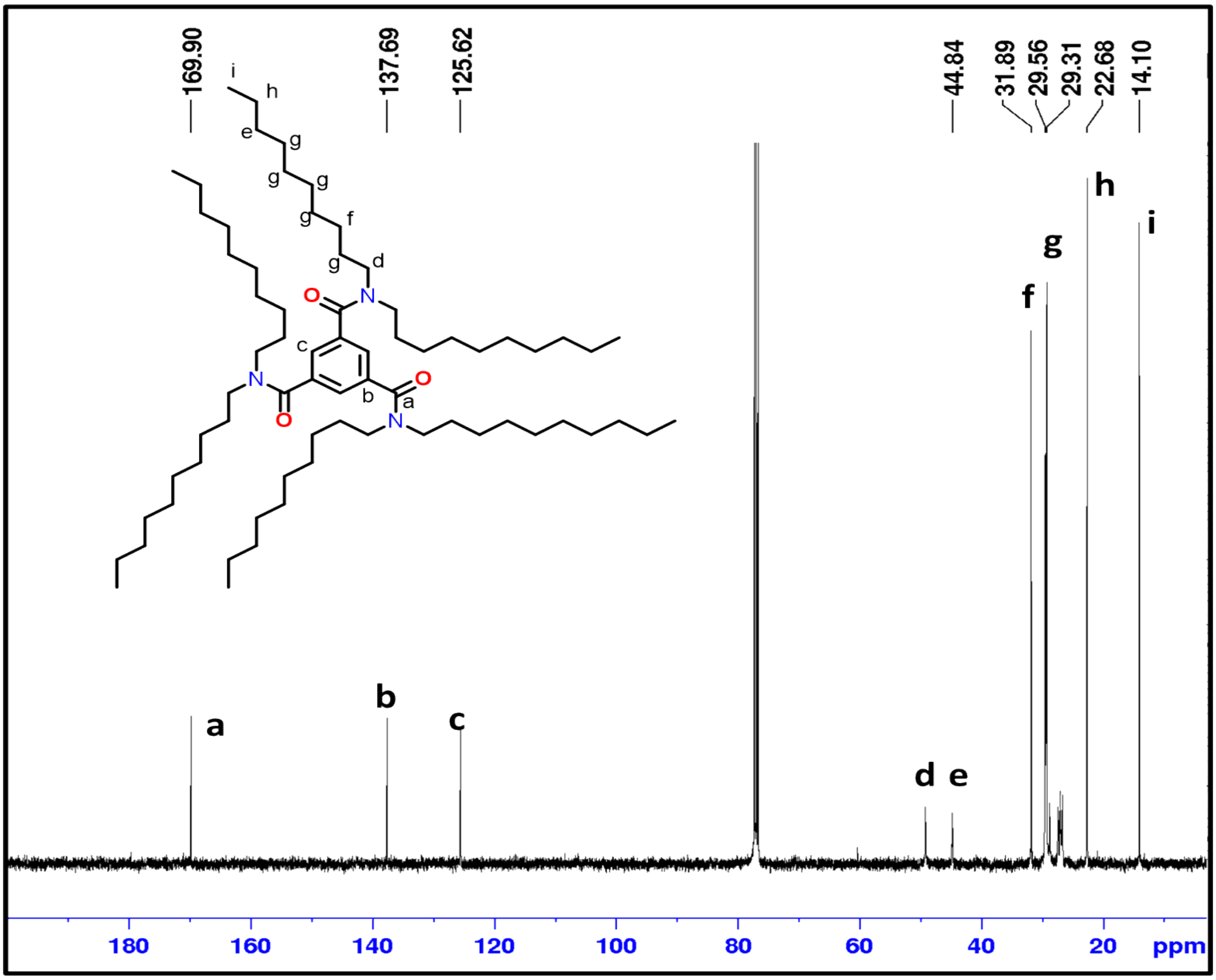

Fig. S11 ^{13}C NMR spectrum of HDBTA

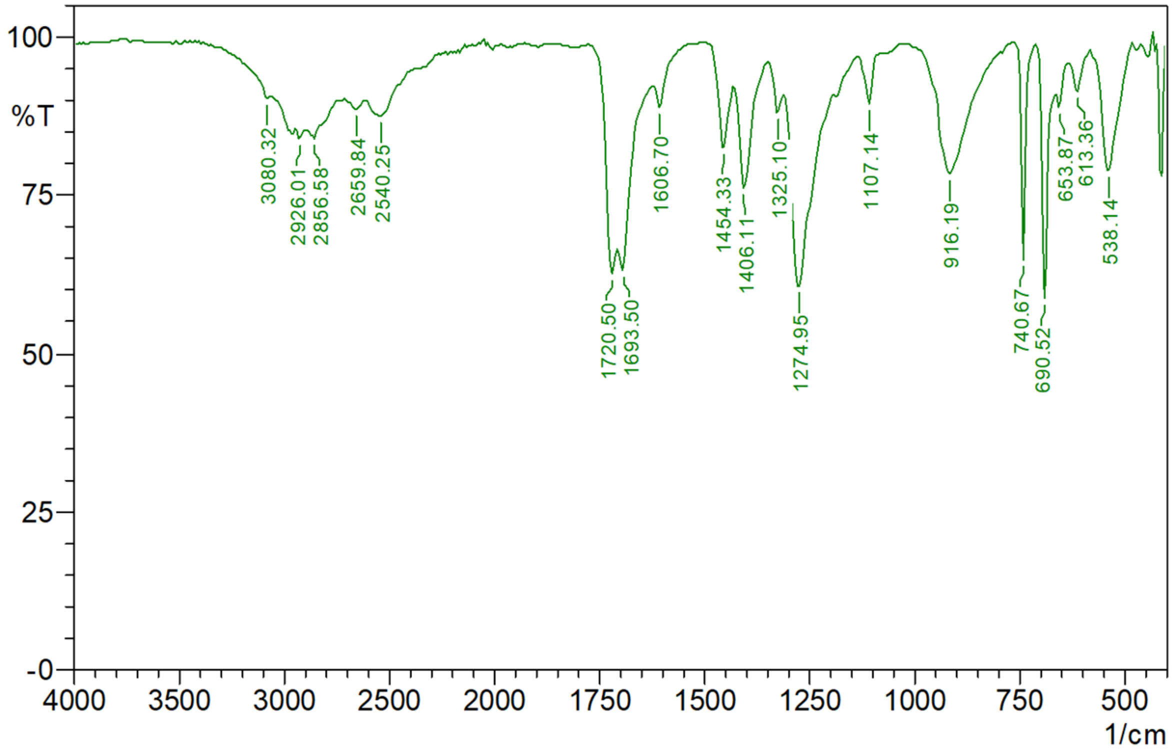

Fig. S12 FT-IR spectrum of HDBTA

Table S1. Exact amount of the modifiers adsorbed onto the monolithic support.

Column Modifier	Initial amount of column modifiers (mmol)	MeOH:H_2O (v/v)	Amount of modifier coated (mmol)
**HBBTA**	0.10	75:25	0.06
	0.25	76:24	0.08
	0.50	78:22	0.13
	**1.50**	**73:27**	**0.17**
**HHBTA**	**0.10**	**78:22**	**0.07**
	0.25	78:22	0.09
	0.50	78:22	0.14
**HOBTA**	0.10	89:11	0.03
	0.20	93:7	0.05
	**0.50**	**92:8**	**0.07**
**HDBTA**	0.01	95:5	0.006
	0.025	93:7	0.012
	**0.1**	**92:8**	**0.023**

Table S2. Optimized chromatographic conditions

HPLC system	JASCO LC 4000 Plus
Column	C_{18} silica monolithic column (RP18e, 100 × 4.6 mm, Merck-Chromolith high-resolution)
Column temperature	Ambient
Column modifier	N^1,N^1,N^3,N^3,N^5,N^5-hexa(butyl)benzene-1,3,5-tricarboxamide (HBBTA)<br>N^1,N^1,N^3,N^3,N^5,N^5-hexa(hexyl)benzene-1,3,5-tricarboxamide (HHBTA)<br>N^1,N^1,N^3,N^3,N^5,N^5-hexa(octyl)benzene-1,3,5-tricarboxamide (HOBTA)<br>N^1,N^1,N^3,N^3,N^5,N^5-hexa(decyl)benzene-1,3,5-tricarboxamide (HDBTA)
Optimum probe content	0.17 mM HBBTA, 0.07 mM HHBTA & HOBTA, 0.023 mM HDBTA
Mobile phase	0.1 M of 2-hydroxyisobutyric acid (α-HIBA, pH 2.5)
Mobile phase flow rate	1 mL/min
Sample volume	20 µL
PCR	0.015 mM of Arsenazo(III) (pH 7.0)
PCR flow rate	1.5 mL/min
Detector	JASCO UV-4070
Wavelength	665 nm
